# Supplementary material for: A cell-and-plasma numerical model reveals hemodynamic stress and flow adaptation in zebrafish microvessels after morphological alteration
Source: PLoS Comput Biol. 2023 Dec 4;19(12):e1011665. doi: 10.1371/journal.pcbi.1011665 (PMC10721208; doi:10.1371/journal.pcbi.1011665)
Supplement: S1 Text — (PDF) [file pcbi.1011665.s001.pdf]

S1 Text. Tables for Simulation and Experiment Data

E_{s0}	3 µN/m	Resting shear modulus: Matched against uniaxial stretch test by optical tweezers [1], validation shown in supplementary material figure S2 of our previous work [2]
E_b	1.2×10^{-19} J	Bending modulus: Matched against aspiration and membrane buckling experiment [3], validation shown in supplementary material figure S3A and S3B of our previous work [2]
$K_0 = 2E_{s0} + k_a + k_d$	116 µN/m	Area compressibility modulus: Relaxed to 1/4000 levels seen in swollen RBC aspiration experiment [3, 4] in order to ensure numerical stability in the CGSM. Following the approach of [5] this property was kept 10x larger than E_{s0} to ensure low areal dilation/compression in the RBC deformations
k_a	77 µN/m	Global area coefficient: Formulation from [5]
k_d	36 µN/m	Local area coefficient: Formulation from [5]
k_Ω	55 N/m^3	Volume penalty coefficient: Formulation from [5]. This property was kept 10x larger than E_{s0} to ensure low volume dilation in the predicted RBC deformations
$u_{repul,RBCtoRBC}$	8.333×10^{-7} m/s	RBC repulsion coefficient: from neighboring RBC membrane surfaces to the RBC node
$r_{sep,RBCtoRBC}{}^*$	1 µm	RBC separation threshold: RBC surfaces in proximity below this separation threshold will experience inter-surface repulsion
$u_{repul,NuctoRBC}$	4.167×10^{-7} m/s	Nucleus repulsion coefficient: from neighboring nucleus nodes to the RBC sub-triangle elements around the RBC node
$r_{sep,NuctoRBC}{}^*$	0.2 µm	Nucleus separation threshold: RBC membrane sub-triangle elements near nucleus nodes below this threshold will experience repulsion
$u_{repul,WalltoRBC}$	1×10^{-4} m/s	Wall repulsion coefficient: from the closest lumen wall point to the RBC node
$r_{sep,WalltoRBC}{}^*$	0.25 µm	Wall separation threshold: If the distance between an RBC membrane node and a lumen wall surface point is lower than this threshold then the membrane node will experience repulsion away from the lumen wall

Table A. RBC material properties and contact dynamics parameters employed in simulations.

Model	Region (refer to Fig 1A)	$P_{diastole}$ [Pa]	P_{range} [Pa]	T [s]	ω_{lag}
WT	CA anterior: CA1	54	82	0.36	0
	CA posterior: CA2	48	68		0.06
	CV anterior: CV3	11	23		0.2
	CV posterior (small): CV2	23	27		0.2
	CV posterior (big): CV1	18	27		0.2
	DLAV anterior: DLAV1	29	66		0.1
	DLAV posterior: DLAV2	28.5	60		0.1
NoRBC1	CA1	33.28	59.04	0.36	0
	CA2	28.96	48.96		0.06
	CV3	2.32	16.56		0.2
	CV2	10.96	19.44		0.2
	CV1	7.36	19.44		0.2
	DLAV1	15.28	47.52		0.1
	DLAV2	14.92	43.2		0.1
NoRBC2	Same conditions as NoRBC1				
SGM1	Same conditions as WT				
SGM2	Same conditions as WT				
SGM3	Same conditions as WT				
ML1OE	Same conditions as WT				
ML1KO1	CA1	37.60456	59.93988	0.46	0
	CA2	32.15548	49.04172		0.06
	CV3	4.13164	14.01192		0.2
	CV2	13.47292	17.12568		0.2
	CV1	9.58072	17.12568		0.2
	DLAV1	18.14356	47.48484		0.1
	DLAV2	16.9759	42.8142		0.1
ML1KO2	CA1	54	77	0.46	0
	CA2	47	63		0.06
	CV3	11	18		0.2
	CV2	23	22		0.2
	CV1	18	22		0.2
	DLAV1	29	61		0.1
	DLAV2	27.5	55		0.1
ML1KO3	CA1	67.5	96.25	0.36	0
	CA2	58.75	78.75		0.06
	CV3	13.75	22.5		0.2
	CV2	28.75	27.5		0.2
	CV1	22.5	27.5		0.2
	DLAV1	36.25	76.25		0.1
	DLAV2	34.375	68.75		0.1
PlxnD1	Same conditions as WT				

Table B. Simulation boundary condition settings.

		Average peak systolic velocity [μm/s]					Time-averaged discharge hematocrit (Hd)				
Data source	No. of embryos at 2dpf	CA	CV	aISVs	vISVs	ISVavg	CA	CV	aISVs	vISVs	ISVavg
Ye et al., 2022 [6]	27	2302.5	1326.5	1002	869	936	16.3	16.8	4.12	4.325	4.223
WT simulation	-	2307	1011	1069	1062	1066	17.80	18.12	4.698	3.298	3.998
Discrepancy [%]		0.20	-23.78	6.69	22.21	13.89	9.20	7.86	14.03	-23.75	-5.33

Table C. Comparison of the wild-type (WT) network flow velocity and vessel discharge hematocrit in the CA, CV, aISVs and vISVs against experimental reference [6].

Group	Fish #	Heart rate [BPM]	peak velocity [µm/s]		
			CA	CV	ISV
Gata1 MO (0.1uM)	1	174	2234	1765	1144
Gata1 MO (0.1uM)	2	174	3921	2582	640
Gata1 MO (0.1uM)	3	186	2722	1997	558
Gata1 MO (0.1uM)	4	156	2934	2447	1025
Gata1 MO (0.1uM)	5	180	2843	1922	1295
Gata1 MO (0.1uM)	6	147	3110	2746	648
Gata1 MO (0.1uM)	7	150	2082	1904	844
Control MO	1	186	2298	1587	1641
Control MO	2	180	2482	1871	1635
Control MO	3	150	2366	1943	1447
Control MO	4	174	2456	1821	1195
Control MO	5	168	2538	1745	982
Control MO	6	144	2295	1431	922
Control MO	7	162	2617	2092	826
Control MO	8	150	2780	2103	1041
Gata1 MO average		167	2835	2195	879
Control MO average		164	2479	1824	1211

Table D. Comparison of RBC flux concentration and flow velocity in *gata1* morpholino-injected 2 dpf zebrafish embryos (Gata1 MO) versus control morpholino (Control MO).

Group	ISV perfusion	Fish #	Heart rate [BPM]	peak velocity [μm/s]			RBC flux per ISV [RBC/s]	Relative change in ISV flux from WT average [%]
				CA	CV	ISV		
WT Control	High	1	168	2268	1621	1322	4.79	-23.9
WT Control	High	2	168	2372	2033	1612	6.8	8.0
WT Control	High	3	156	2239	1708	1189	8.12	28.9
WT Control	High	4	156	2033	1672	1365	5.48	-13.0
WT Control	Moderate	5	162	2691	1571	980	0.88	-86.0
Marcksl1 KO	High	6	162	2084	1865	1297	5.22	-17.1
Marcksl1 KO	High	10	156	2349	1830	1968	6.42	1.9
Marcksl1 KO	High	13	138	2713	1959	1643	5.29	-16.0
Marcksl1 KO	High	15	138	2622	1969	1659	6.14	-2.5
Marcksl1 KO	Moderate	1	138	2341	1687	1041	2.24	-64.4
Marcksl1 KO	Moderate	2	144	2035	1933	1308	3.33	-47.1
Marcksl1 KO	Moderate	12	138	2229	1280	951	1.98	-68.6
Marcksl1 KO	Moderate	3	162	2443	2058	1094	1.2	-80.9
Marcksl1 KO	Moderate	7	140	1928	1743	1474	1.11	-82.4
Marcksl1 KO	Moderate (CA defect)	9	140	1351	876	1457	1.05	-83.3
Marcksl1 KO	Low	5	140	1073	910	411	0.05	-99.2
Marcksl1 KO	Low	8	126	1701	1186	651	0.16	-97.5
Marcksl1 KO	Low	14	120	941	497	509	0.04	-99.4
Marcksl1 KO	None	4	150	680	1169	0	0	-100.0
Marcksl1 KO	None	11	144	0	0	0	0	-100.0

Table E. List of flow velocity and RBC perfusion level in the ISV networks amongst the Marcksl1 KO and WT zebrafish embryos at 2 dpf from experiments.

Group	Heart rate [BPM]	peak velocity [µm/s]			RBC flux per ISV [RBC/s]	Relative change in ISV flux from WT average [%]
		CA	CV	ISV		
**WT expt. high group average**	**162**	**2228**	**1759**	**1372**	**6**	**-**
WT simulation	167	2306	1011	1066	3.49	-
WT model discrepancy [%]	3.1	3.5	-42.5	-22.3	-44.6	-
**Marcksl1KO expt. low & moderate group average**	**138.5**	**1836**	**1412**	**930**	**1.26**	**-79.9**
ML1KO1 simulation	130	1445	581	477	0.43	-93.2
ML1KO1 discrepancy [%]	***-6.1***	***-21.3***	***-58.8***	***-48.7***	***-66.0***	***16.6***
ML1KO2 simulation	130	1923	770	804	0.67	-89.4
ML1KO2 discrepancy [%]	***-6.1***	***4.7***	***-45.5***	***-13.5***	***-47.0***	***11.8***
ML1KO3 simulation	167	2389	944	929	0.54	-91.4
ML1KO3 discrepancy [%]	***20.6***	***30.1***	***-33.1***	***-0.1***	***-57.3***	***14.4***

Table F. Comparison of average flow velocity and RBC perfusion level in the experiment-observed ISV networks in the WT and low perfusion Marcksl1 KO zebrafish embryos at 2 dpf against the WT model, Marcksl1 KO models 1, 2 and 3 in the simulations.

References

1. Suresh S, Spatz J, Mills JP, Micoulet A, Dao M, Lim CT, et al. Connections between single-cell biomechanics and human disease states: gastrointestinal cancer and malaria. Acta Biomater. 2005;1: 15–30. doi:10.1016/j.actbio.2004.09.001

2. Ye SSM, Kim S. A mechanistic model of cross-bridge migration in RBC aggregation and disaggregation. Frontiers Bioeng Biotechnology. 2022;10: 1049878. doi:10.3389/fbioe.2022.1049878

3. Evans EA. Bending elastic modulus of red blood cell membrane derived from buckling instability in micropipet aspiration tests. Biophys J. 1983;43: 27–30. doi:10.1016/s0006-3495(83)84319-7

4. Evans EA, Waugh R, Melnik L. Elastic area compressibility modulus of red cell membrane. Biophys J. 1976;16: 585–595. doi:10.1016/s0006-3495(76)85713-x

5. Fedosov DA, Pan W, Caswell B, Gompper G, Karniadakis GE. Predicting human blood viscosity in silico. Proc National Acad Sci. 2011;108: 11772–11777. doi:10.1073/pnas.1101210108

6. Ye SSM, Kim JK, Carretero NT, Phng L-K. High-Throughput Imaging of Blood Flow Reveals Developmental Changes in Distribution Patterns of Hemodynamic Quantities in Developing Zebrafish. Front Physiol. 2022;13: 881929. doi:10.3389/fphys.2022.881929
