# Supplementary material for: A cell-and-plasma numerical model reveals hemodynamic stress and flow adaptation in zebrafish microvessels after morphological alteration
Source: PLoS Comput Biol. 2023 Dec 4;19(12):e1011665. doi: 10.1371/journal.pcbi.1011665 (PMC10721208; doi:10.1371/journal.pcbi.1011665)
Supplement: S2 Text — (PDF) [file pcbi.1011665.s002.pdf]

S2 Text. Supplementary text for A cell-and-plasma numerical model reveals hemodynamic stress and flow adaptation in zebrafish microvessels after morphological alteration.

Section A: Relative CA hematocrit and CA flow rate estimation

The relative CA hematocrit ($H_{rel,CA}$) presented in main figures 3A to 3F is the RBC-count ($\dot{N}_{rbc}$) divided by average lumen cross-sectional area (A_c) and normalized against the maximum level seen in control morpholino injected embryos (H_{max}) (Control MO#3 in Fig. Aviii):

$$H_{rel} = \frac{\dot{N}_{rbc}}{A_c} / H_{max} \qquad (A1a)$$

$$where \;\; H_{max} = \frac{\dot{N}_{rbc,controlMO\#3}}{A_{c,controlMO\#3}} \qquad (A1b)$$

The CA flow rate was estimated by multiplying the lumen bulk flow velocity (V_{bulk}) in the CA by the lumen cross-sectional area (A_c):

$$Q = V_{bulk} A_c \qquad (A2a)$$

$$where \;\; V_{bulk} = \varphi V_{centerpeak} \qquad (A2b)$$

$V_{centerpeak}$ is the lumen centerline velocity that we measured from RBC tracking experiments. Since we could not obtain the radial velocity distribution profile using RBC tracking, we had to assume the relationship function between V_{bulk} and $V_{centerpeak}$ given by their ratio φ. φ varies from 0.5 in a Poiseuille flow scenario and 1 in an idealized perfect plug flow scenario. Basically, pure plasma flow where blood is zero hematocrit posits φ values of 0.5 and practical blood flows with varying hematocrit will range from 0.5 to 1. Based on the CFD model of a 30µm-diameter straight lumen segment in section 2.1, φ was determined to be 0.64 at 20% hematocrit condition for a CA-sized lumen. Scaling this linearly for 14% hematocrit (this is the average hematocrit for CA in the anterior caudal region at 2 dpf as reported by our reference [1]) we found φ value to be 0.59. Control MO#3 was assumed to represent 14% hematocrit level while the other samples were scaled linearly for φ as a function of the relative CA hematocrit:

$$\varphi = 0.5 + H_{rel}(0.59 - 0.5) \qquad (A3)$$

Section B: Experiment flow data of trunk vessels from *gata1* and control morpholino injected embryos at 2 dpf

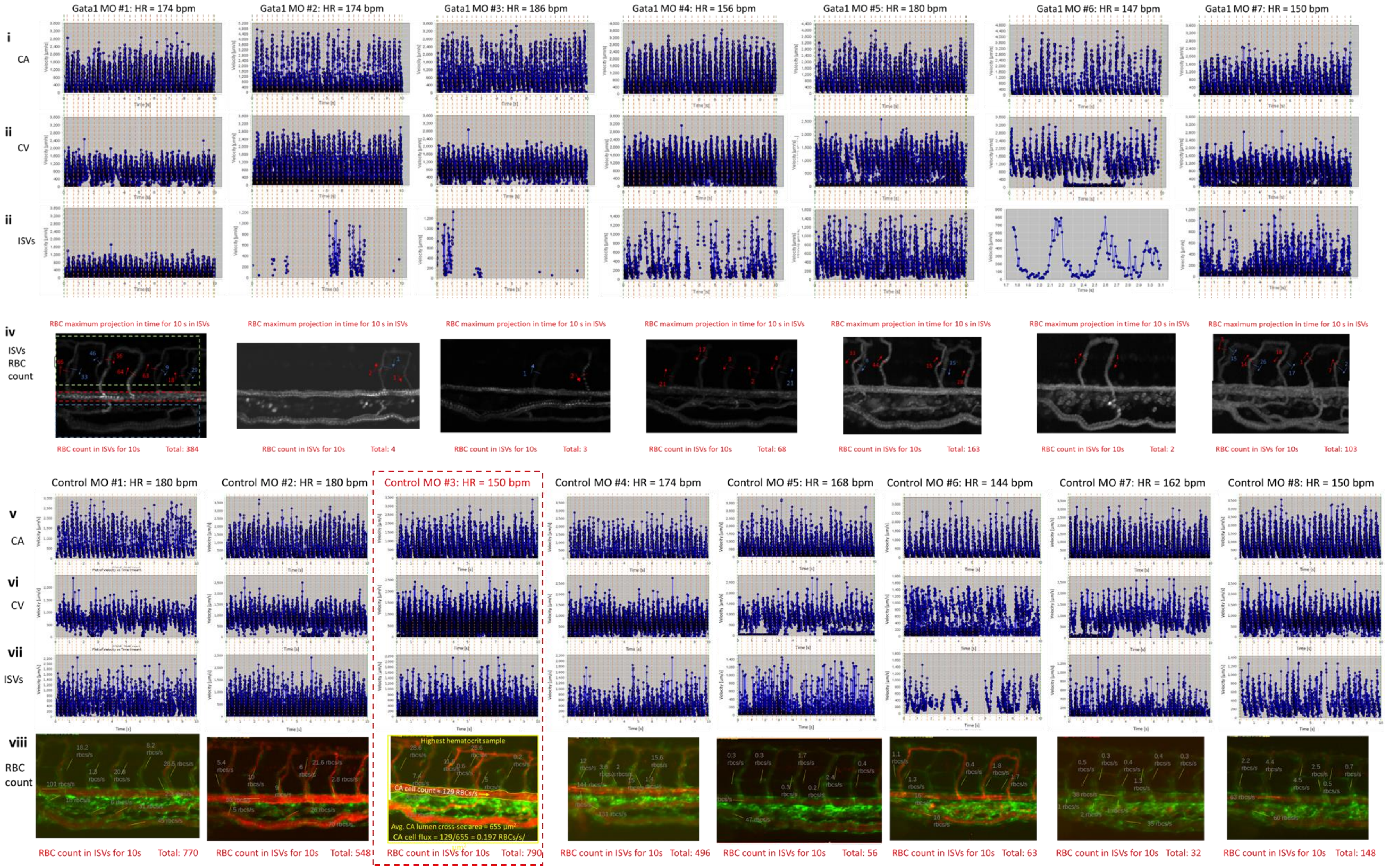

Fig A. RBC velocity data in the caudal artery CA **(i)**, caudal vein CV **(ii)** and intersegmental vessels ISVs **(iii)** in trunk region pertaining to the simulation model domain (posterior to the yolk extension) for gata1 morphants at 2dpf and their corresponding RBC counts in the ISVs **(iv)**. RBC velocity data in the CA **(v)**, CV **(vi)** and ISVs **(vii)** in trunk region posterior to the yolk extension for control morpholino injected embryos at 2dpf and the corresponding RBC counts into ISVs **(viii)**. Orange grid lines in velocity plots (i to iii, v to vii) indicate the time positions of systolic peaks in the CA velocity and show the phase lag between CA peaks and CV peaks. Red-dashed box in (v to viii) highlight Control MO #3 zebrafish, which was used as the normalization reference for calculating the relative CA hematocrit in both gata1 MO and control MO groups.

Section C: Experiment flow data of trunk vessels from wildtype (WT) control and *marcksl1a* and *marcksl1b* double knockout (ML1dbl KO) embryos at 2 dpf

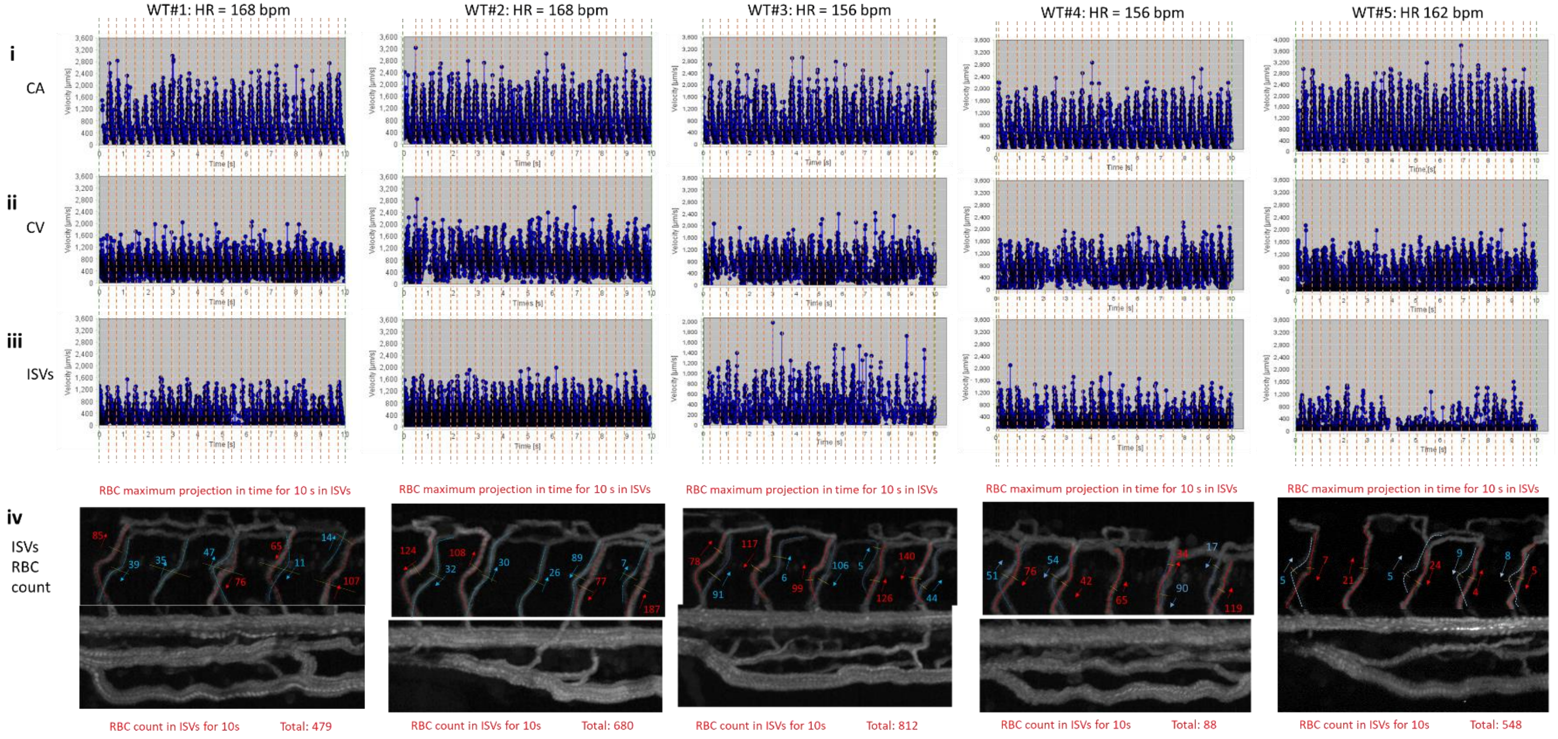

Fig B. RBC velocity data in the caudal artery CA **(i)**, caudal vein CV **(ii)** and intersegmental vessels ISVs **(iii)** in trunk region pertaining to the simulation model domain (posterior to the yolk extension) for WT zebrafish at 2dpf and their corresponding RBC counts in the ISVs **(iv)**. Orange grid lines in velocity plots (i to iii) indicate the time positions of systolic peaks in the CA velocity and show the phase lag between CA peaks and CV peaks.

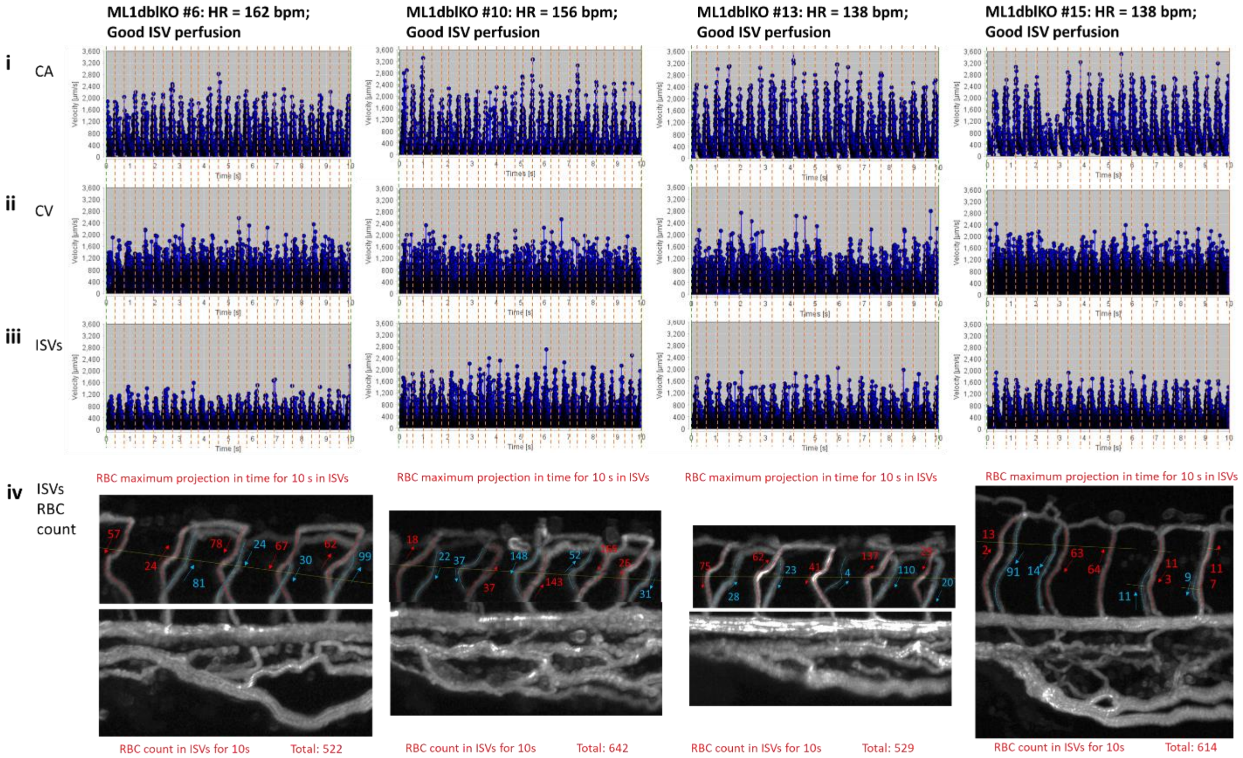

Fig C. RBC velocity data in the caudal artery CA **(i)**, caudal vein CV **(ii)** and intersegmental vessels ISVs **(iii)** in trunk region pertaining to the simulation model domain (posterior to the yolk extension) for Marcksl1a and Marcks1b double knock-out (referred to as Marcksl1KO in main text) zebrafish exhibiting indistinguishable flow and morphological difference from WT at 2dpf. Their corresponding RBC counts in the ISVs are shown in **(iv)**. Orange grid lines in velocity plots (i to iii) indicate the time positions of systolic peaks in the CA velocity and show the phase lag between CA peaks and CV peaks.

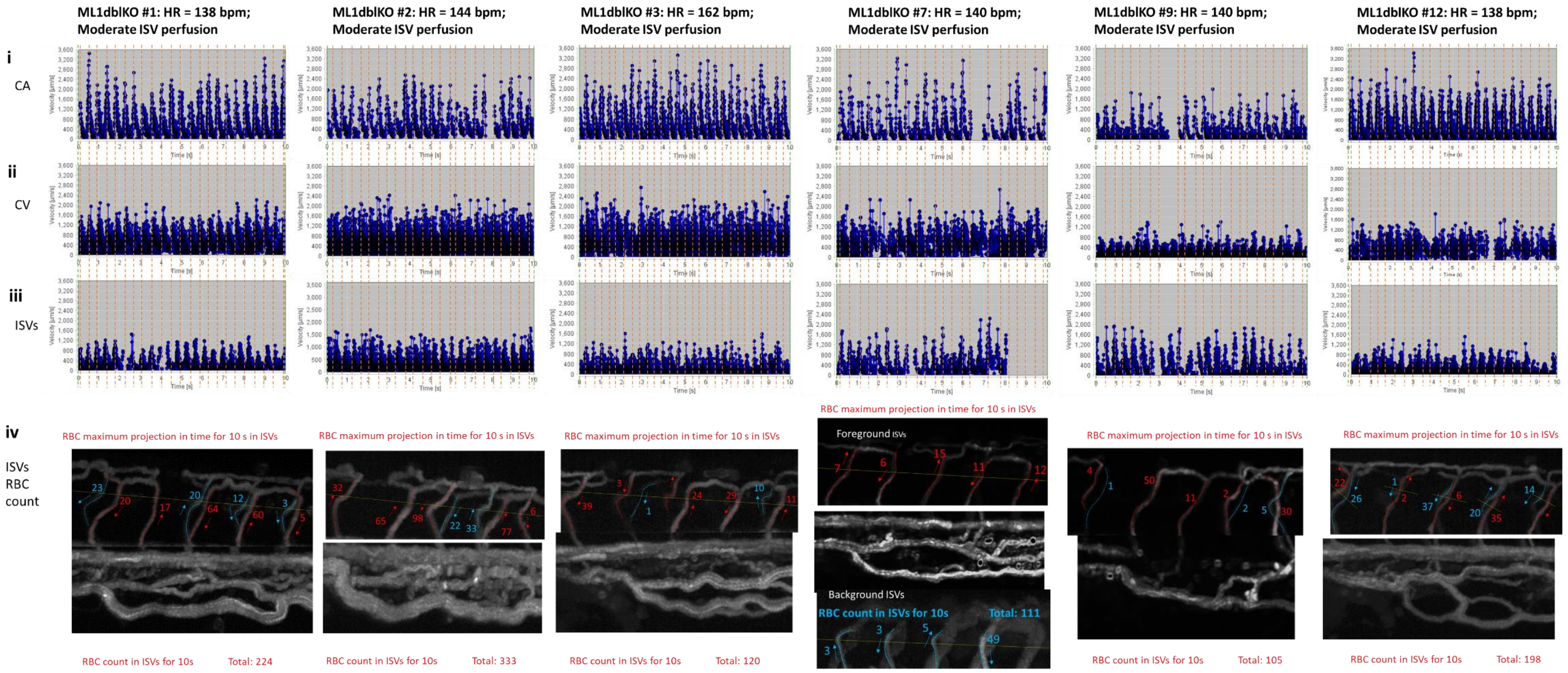

Fig D. RBC velocity data in the caudal artery CA **(i)**, caudal vein CV **(ii)** and intersegmental vessels ISVs **(iii)** in trunk region pertaining to the simulation model domain (posterior to the yolk extension) for Marcksl1a and Marcks1b double knock-out (referred to as Marcksl1KO in main text) zebrafish exhibiting moderate lumen diameter and RBC perfusion reduction phenotyping at 2dpf. Their corresponding RBC counts in the ISVs are shown in **(iv)**. Orange grid lines in velocity plots (i to iii) indicate the time positions of systolic peaks in the CA velocity and show the phase lag between CA peaks and CV peaks.

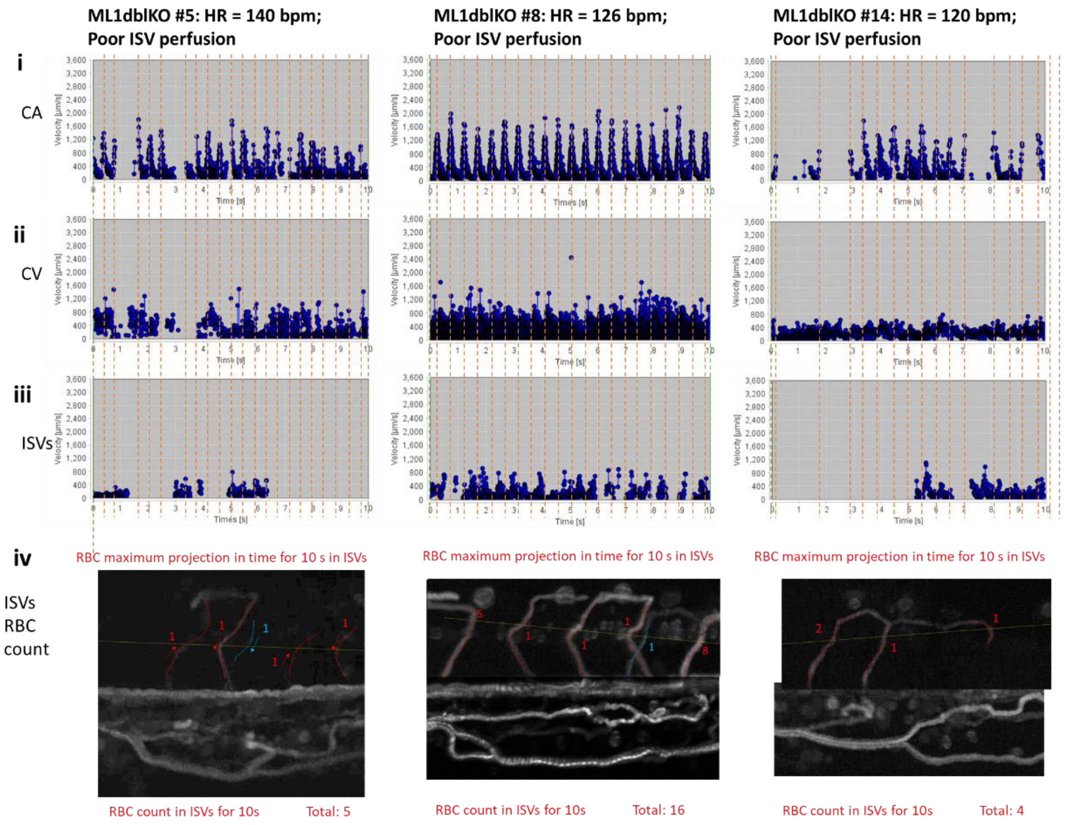

Fig E. RBC velocity data in the caudal artery CA **(i)**, caudal vein CV **(ii)** and intersegmental vessels ISVs **(iii)** in trunk region pertaining to the simulation model domain (posterior to the yolk extension) for Marcksl1a and Marcks1b double knock-out (referred to as Marcksl1KO in main text) zebrafish exhibiting extensive lumen diameter and RBC perfusion reduction phenotyping at 2dpf. Their corresponding RBC counts in the ISVs are shown in **(iv)**. Orange grid lines in velocity plots (i to iii) indicate the time positions of systolic peaks in the CA velocity and show the phase lag between CA peaks and CV peaks.

The CA flow rate in the Marcksl1 KO experiments plotted in main figures 8E and 8F were estimated using Eqs (A1) – (A3).

Section D: Supplementary data for smooth geometry model networks comparison

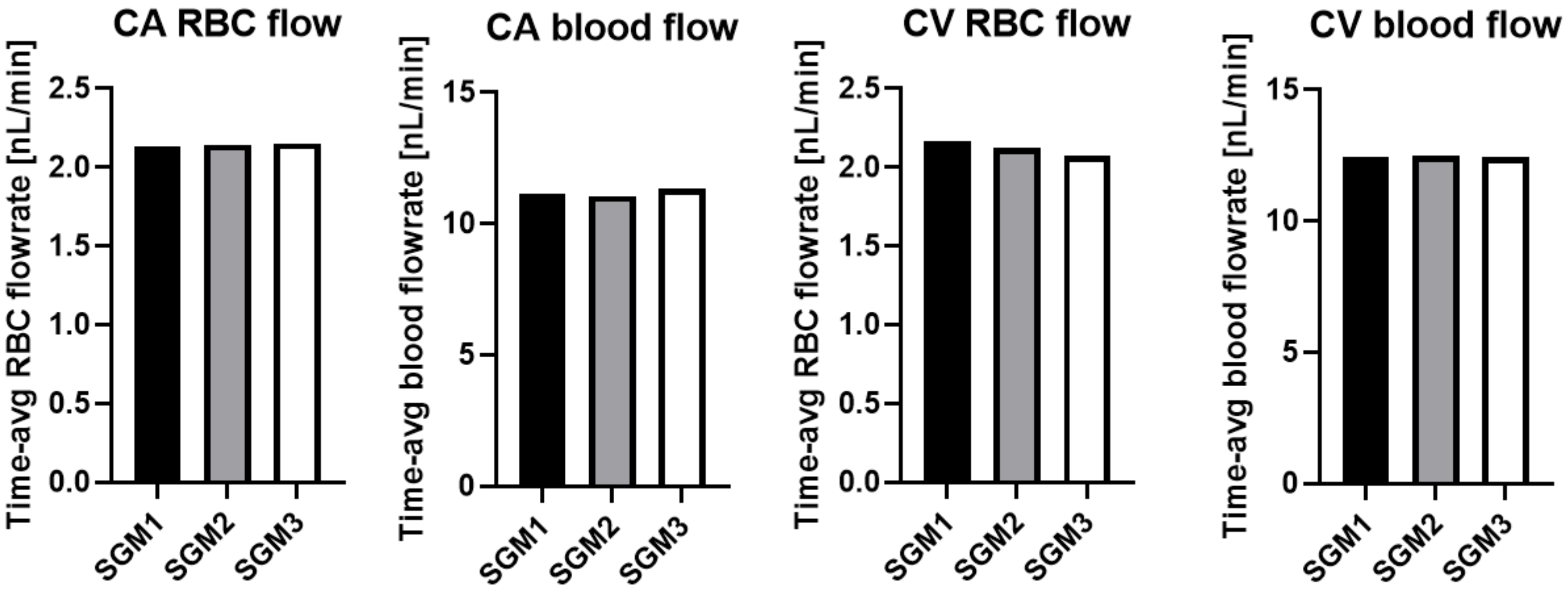

Fig F. RBC flow rate and blood flow rate comparisons between smooth geometry models (SGMs) for the caudal artery (CA) and caudal vein (CV) vessels. The results indicate the same flow-rate condition setting in the CA and CV amongst the 3 SGMs: 2.13, 2.14, 2.15 nL/min for RBC flow rate and 11.11, 11.04, 11.33 nL/min for blood flow rate in the CA for SGM1, SGM2 and SGM3; 2.07, 2.12, 2.16 nL/min for RBC flow rate and 12.45, 12.48, 12.44 nL/min for blood flow rate in the CV for SGM1, SGM2 and SGM3,.

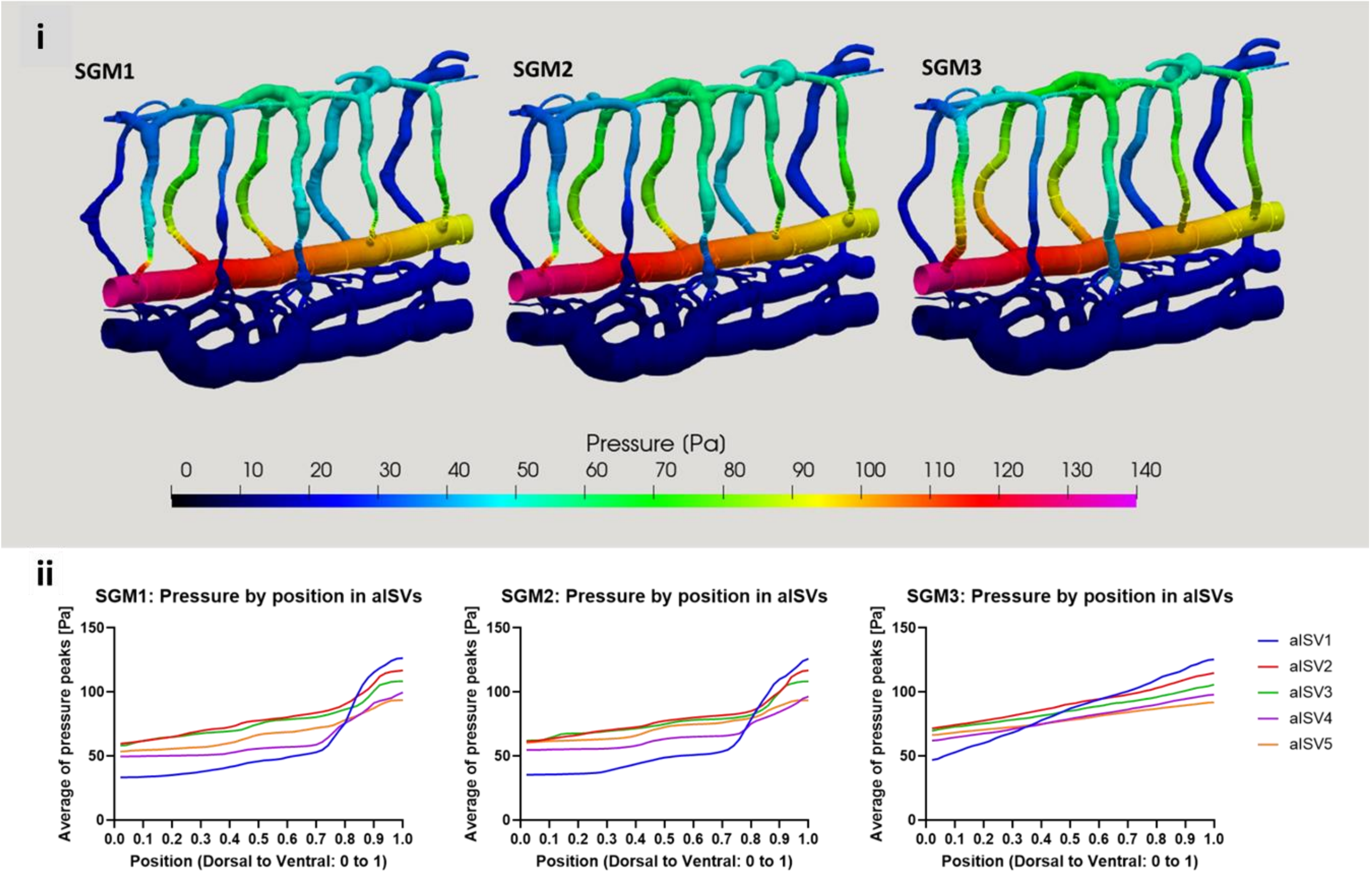

Fig G. Pressure distributions for the 3 SGMs **(i)** indicating pressure gradient accentuation in ventral regions of aISVs in SGM1 and SGM2 due to the narrowing of lumen cross-sections in the dorsal to ventral direction , in contrast to smooth pressure gradients in aISVs of SGM3 that has constant lumen diameters throughout the vessel length **(ii)**

Section E: Supplementary data for Marcksl1 mutant model networks comparison

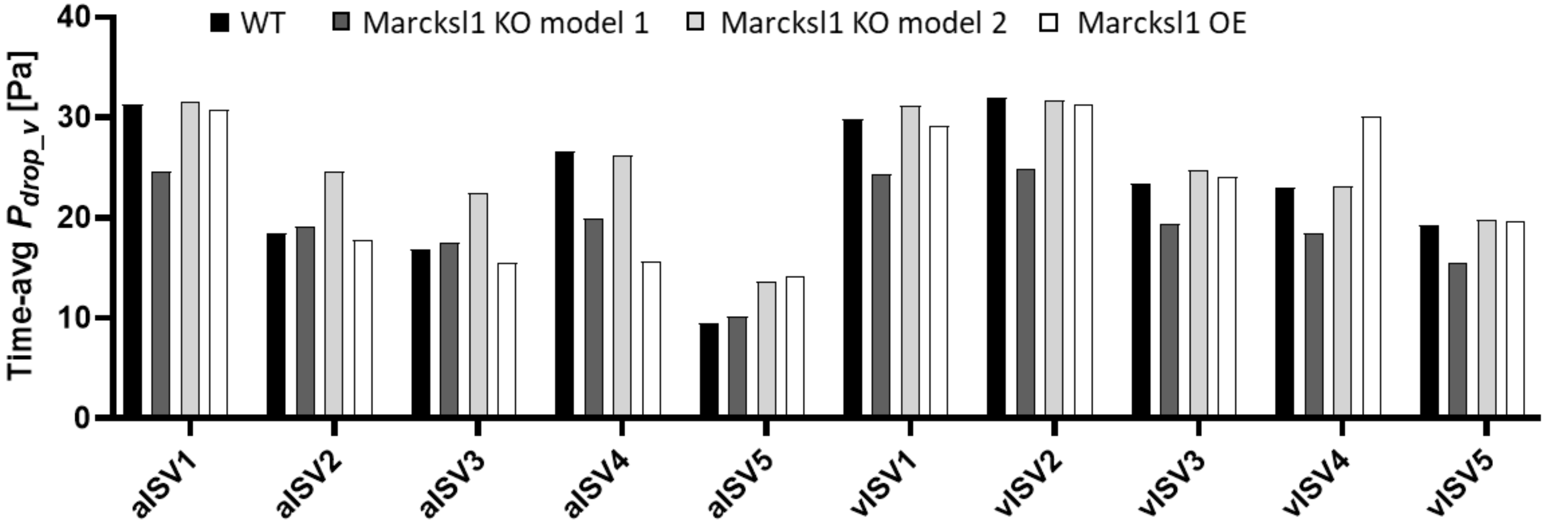

Fig H. Pressure drop comparisons amongst the Marcksl1 mutant network models against WT network model for individual 10 intersegmental vessels (ISVs) in the modelled domains. The results show that network-level alterations in morphology for Marcksl1 mutants redistribute pressure distributions in the network compared to WT network levels. The morphology-induced pressure redistribution further compounded the effects of altered systemic flow and pressure conditions discussed in the main paper.

Section F: The coarse-grained spectrin model (CGSM) of RBC mechanics

The zebrafish RBC is modelled as a flattened ellipsoid capsule with an RBC membrane shell enveloping a cytosolic interior and a nucleus. The membrane is represented by a hexagonal mesh where edges represent the cytoskeleton (CSK) of spectrin chains joined to actin filaments [2,3] and the triangle surface elements represent the plasma membrane (PM). Likewise, the nucleus is defined as a three-dimensional meshwork where enjoining edges act as compressible springs for the prescription of nuclear deformation. Tension in the RBC membrane arising from deformation consists of the elastic component and the viscous component:

$$\boldsymbol{F}_{m,i} = \boldsymbol{F}_{visc,i} + \boldsymbol{F}_{elastic,i} \qquad (F1)$$

where $\boldsymbol{F}_{visc,i}$ is the viscous force and $\boldsymbol{F}_{elastic,i}$ is the elastic force at membrane vertex i. The elastic force may be obtained by differentiating the local elastic energy ($U_{elastic,i}$) with respect to the nodal displacement ($\boldsymbol{s_i}$) produced by the deformation:

$$\boldsymbol{F}_{elastic,i} = \frac{\partial U_{elastic,i}}{\partial \boldsymbol{s_i}} \qquad (F2)$$

The elastic constitution of the RBC membrane can be decomposed to the individual mechanical aspects of the PM and CSK:

$$U_{elastic} = U_{spring} + U_{bend} + U_{area} + U_{volume} \qquad (F3)$$

Shear elasticity of the CSK and stiffness of the RBC nucleus

Shearing properties of the CSK were modelled using the worm-like chain (WLC) extension model and the power-law (POW) compression model (Fedosov et al., 2010a):

$$U_{spring} = \sum_{ij\in(1\ldots N_e)} U_{WLC}(l_{ij}) + \sum_{ij\in(1\ldots N_e)} U_{POW}(l_{ij}) \qquad (F4)$$

$$U_{WLC} = k_B T\left(\frac{l_m}{4p}\right)\left(\frac{3\left(\frac{l_{ij}}{l_m}\right)^2 - 2\left(\frac{l_{ij}}{l_m}\right)^3}{1-\left(\frac{l_{ij}}{l_m}\right)}\right) \qquad where\ 0 < \frac{l_{ij}}{l_m} < 1 \qquad (F5)$$

$$U_{POW} = \frac{k_p}{l_{ij}} \qquad (F6)$$

where l_{ij} is the mesh edge length between vertices i and j, l_m is the maximum allowable edge length in the WLC model and p is the persistence length. $k_B T$ is the energy per unit mass which may be considered as a membrane constant for an isothermal consideration. k_p is the spring constant for the POW compression spring model. The resting shear modulus (E_{s0}) of the WLC-POW network can be given by the following relation[4]:

$$E_{s0} = \left(\frac{\sqrt{3}k_B T}{4pl_{ij,0}}\right)\left(\frac{\frac{l_{ij,0}}{l_m}}{2\left(1-\frac{l_{ij,0}}{l_m}\right)^3} - \frac{1}{4\left(1-\frac{l_{ij,0}}{l_m}\right)^2} + \frac{1}{4}\right) + \frac{3\sqrt{3}k_p}{4{l_{ij,0}}^3} \qquad (F7)$$

where $l_{ij,0}$ is the mesh edge length for the RBC at rest.

Using the WLC-POW springs we are able to exhibit the strain hardening behavior of RBC membranes (see Fig. 10A). We employed the same WLC-POW spring elements for the discretized network of springs that model the stiffness of the RBC nucleus.

Bending elasticity

The bending energy in the mesh network was calculated using the angle θ_{ij} subtended by the normals ($\frac{\vec{\xi}_1}{|\vec{\xi}_1|}$ and $\frac{\vec{\xi}_2}{|\vec{\xi}_2|}$) of the two adjoining triangular mesh elements along their shared mesh edge *ij*:

$$U_{bend} = \sum_{ij\in(1\ldots N_e)} \frac{2}{\sqrt{3}} E_b\left[1 - cos\left(\theta_{ij} - \theta_{ij,0}\right)\right] \qquad (F8)$$

where E_b is the bending modulus, $\theta_{ij,0}$ is the local spontaneous angle that a free sheet of spectrin polymers will adopt in their unstressed state. Since we assume the RBC rest state to be the reference energy state in our model, $\theta_{ij,0}$ is the angle between triangular mesh elements for the RBC at rest.

Area elasticity

The PM has been reported to be highly incompressible and area dilations exceeding 2-4 % produce immediate lysis [5,6]. Incompressibility of the PM was prescribed by the area penalty model:

$$U_{area} = \frac{k_d\left(A_{RBC} - A_{RBC,0}\right)^2}{2A_{RBC,0}} + \sum_{tri\in(1\ldots N_{tri})} \frac{k_a\left(A_{tri} - A_{tri,0}\right)^2}{2A_{tri,0}} \qquad (F9)$$

where the area deformation energy is a combination of both global and local surface area changes. A_{tri} is the current area of the local triangular mesh element and $A_{tri,0}$ is the area of the element when the RBC is at rest. A_{RBC} is the current surface area of the RBC membrane and $A_{RBC,0}$ is the membrane surface area of the RBC at rest. k_d represents the global area compressibility coefficient while k_a is the local compressibility coefficient. The compressibility coefficients may be related to the area compressibility modulus by the following relation [4]:

$$\mathrm{K} = 2E_{s0} + k_a + k_d \qquad \mathrm{(F10)}$$

As K (0.432 N/m) is much larger than E_{s0} (7 µN/m), it is practically determined by the $k_a + k_d$ contribution in the model. For simplicity in the model, k_a and k_d were set to equal values.

Volume constraint

The volume incompressibility is enforced to ensure the RBC volume is conserved. This is achieved by an isotropic penalty term similar to the area incompressibility energy function:

$$U_{volume} = \frac{k_\Omega(\Omega - \Omega_0)^2}{2\Omega_0} \qquad (F11)$$

where Ω is the current cytoplasmic volume of the RBC, Ω_0 is the original cytoplasmic volume based on the mean corpuscular volume (MCV) of 100 fL and k_Ω is the volume correction penalty coefficient set as 220 N/m^3.

Section G: Plasma and cytosol transport model: Lattice Boltzmann Method (LBM)

The LBM solves the Boltzmann equation numerically by discretizing the mass and momentum of fluid particles on a fixed computational lattice. In the LBM, the behavior of the macro-system (defined by pressure, viscosity and density) is given by the kinetics and sum of its microstates. Microstates are defined in the discrete packets by the density distribution function ($f_i(\boldsymbol{x})$) and the discretized transport equation involves the streaming and collision of $f_i(\boldsymbol{x})$ in the lattice. In the streaming process, 19 discrete streaming directions and lattice velocities are defined as follows [7]:

$$\begin{aligned}
&stationary\text{: } c_0 = 0\\
&xyz\text{: } c_1 = \left(\frac{\Delta x}{\Delta t},0,0\right);\ c_2 = \left(-\frac{\Delta x}{\Delta t},0,0\right);\ c_3 = \left(0,\frac{\Delta x}{\Delta t},0\right);\ c_4 = \left(0,-\frac{\Delta x}{\Delta t},0\right);\ c_5 = \left(0,0,\frac{\Delta x}{\Delta t}\right);\ c_6 = \left(0,0,-\frac{\Delta x}{\Delta t}\right)\\
&xy\ diagonals\text{: } c_7 = \left(\frac{\Delta x}{\Delta t},\frac{\Delta x}{\Delta t},0\right);\ c_8 = \left(-\frac{\Delta x}{\Delta t},-\frac{\Delta x}{\Delta t},0\right);\ c_9 = \left(\frac{\Delta x}{\Delta t},-\frac{\Delta x}{\Delta t},0\right);\ c_{10} = \left(-\frac{\Delta x}{\Delta t},\frac{\Delta x}{\Delta t},0\right)\\
&zy\ diagonals\text{: } c_{11} = \left(0,\frac{\Delta x}{\Delta t},\frac{\Delta x}{\Delta t}\right);\ c_{12} = \left(0,-\frac{\Delta x}{\Delta t},-\frac{\Delta x}{\Delta t}\right);\ c_{13} = \left(0,\frac{\Delta x}{\Delta t},-\frac{\Delta x}{\Delta t}\right);\ c_{14} = \left(0,-\frac{\Delta x}{\Delta t},\frac{\Delta x}{\Delta t}\right)\\
&zx\ diagonals\text{: } c_{15} = \left(\frac{\Delta x}{\Delta t},0,\frac{\Delta x}{\Delta t}\right);\ c_{16} = \left(-\frac{\Delta x}{\Delta t},0,-\frac{\Delta x}{\Delta t}\right);\ c_{17} = \left(\frac{\Delta x}{\Delta t},0,-\frac{\Delta x}{\Delta t}\right);\ c_{18} = \left(-\frac{\Delta x}{\Delta t},0,\frac{\Delta x}{\Delta t}\right) \qquad (G1)
\end{aligned}$$

where Δx is the space step size in the lattice, Δt is the time step size, and symbol *i* denotes the flux direction index. The LB equation with a general force term can be expressed as [8]:

$$f_i(\boldsymbol{x} + \boldsymbol{c_i}\Delta t, t + \Delta t) - f_i(\boldsymbol{x},t) = -\frac{\left|f_i(\boldsymbol{x},t) - f_i^{eq}(\boldsymbol{x},t)\right|}{\tau} + \Delta t\boldsymbol{B}_i \qquad (G2)$$

where $\boldsymbol{B}_i$ is the body force term represented on the microstate and τ is a relaxation parameter towards the equilibrium distribution ($f_i^{eq}(\vec{x},t)$) which can be obtained through:

$$f_i^{eq}(\boldsymbol{x},t) = w_i\,\rho\left[1 + \frac{\boldsymbol{c_i}\cdot\boldsymbol{u}}{c_s{}^2} + \frac{1}{2}\frac{(\boldsymbol{c_i}\cdot\boldsymbol{u})^2}{c_s{}^4} - \frac{1}{2}\frac{|\boldsymbol{u}|^2}{c_s{}^2}\right] \qquad (G3)$$

where ρ is the fluid density and $\boldsymbol{u}$ is the velocity; the two are calculated by the equivalency of the macroscopic state to the sum of microstates: $\rho = \sum_i f_i$, $\boldsymbol{u} = \sum_i f_i\, \boldsymbol{c_i}/\rho$, and $c_s = \frac{\Delta x}{\Delta t}/\sqrt{3}$ (speed of sound in the lattice). The weights, w_i, used in Eq (G3) are given by $w_0 = \frac{1}{3}$, $w_i = \frac{1}{18}$ for $i = 1-6$ and $w_i = \frac{1}{36}$ for $i = 7-18$.

The microstate body force term in Eq (G2) is given by:

$$\boldsymbol{B}_i = \left(1 - \frac{1}{2\tau}\right)\,\omega_i\left[\frac{\boldsymbol{c_i} - \boldsymbol{u}}{c_s} + \frac{\boldsymbol{c_i}\cdot\boldsymbol{u}}{c_s}\boldsymbol{c_i}\right]\cdot\boldsymbol{F}_f \qquad (G4)$$

where $\boldsymbol{F}_f$ is the fluid-structure interaction body force acting on the fluid from the IBM coupling with the RBC membrane solver. Proof of the equivalency between the LBM discretization and the continuum Navier-Stokes equations has been given through the Chapman-Enskog expansion of the LB equation [9] The pressure (P) and kinematic viscosity (ν) of the fluid is represented in the LBM system by:

$$\rho = \frac{P}{c_s^2};\quad \tau = \frac{1}{2} + \frac{\nu}{c_s{}^2\Delta t} \qquad (G5)$$

Section H: Boundary condition sensitivity issues with Lattice-Boltzmann and Immersed Boundary Solver Techniques

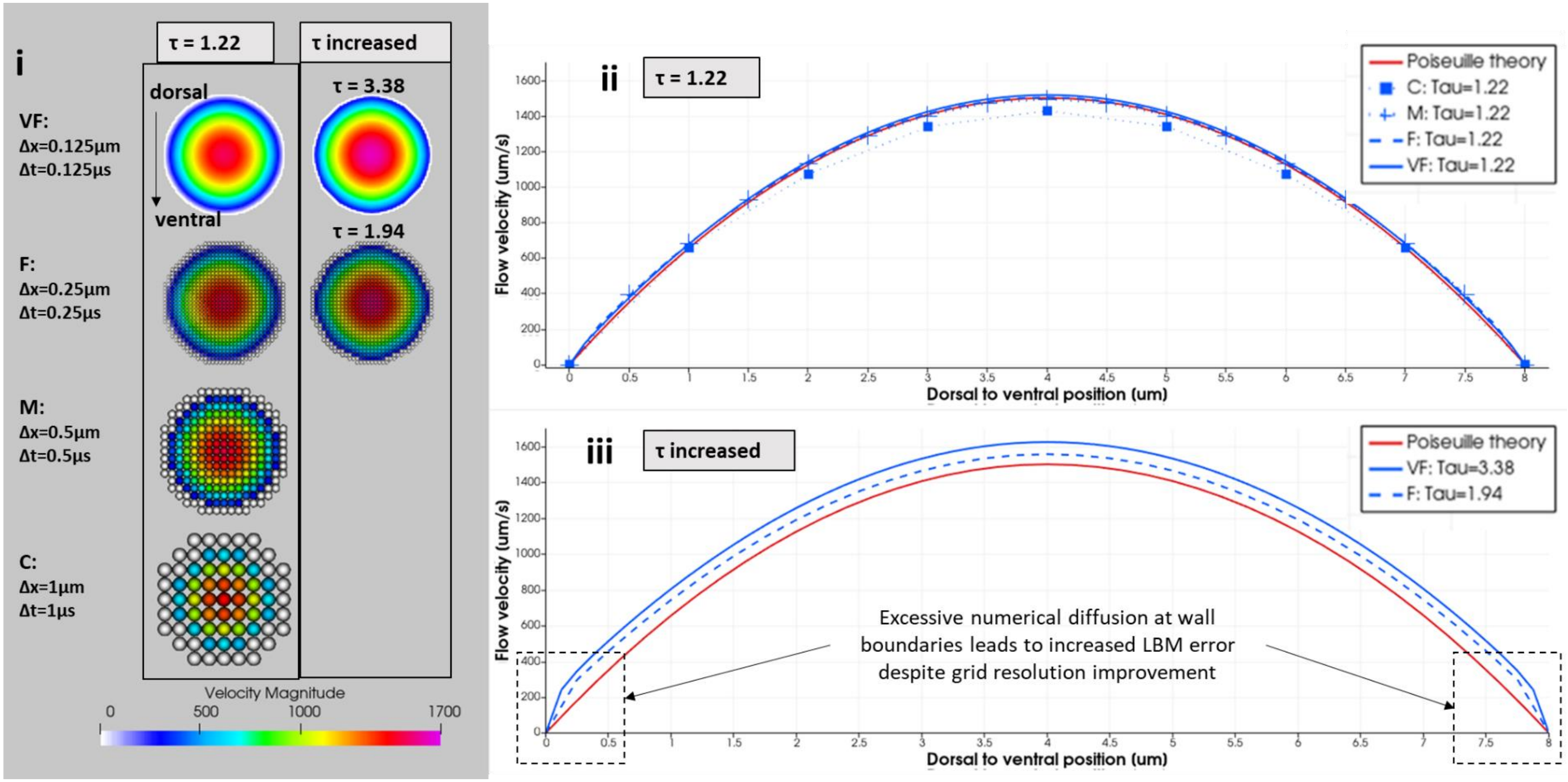

Fig I. The velocity prediction of the LBM solver for straight circular tube of 30 µm and 150 µm in length, performed at different grid resolutions and different relaxation parameter (τ) values. **(i)** Velocity map fills of the lumen cross-section and **(ii and iii)** parabolic velocity profiles in the dorsal to ventral direction of the cross-sectional lumen plane at different LBM grid and τ settings. τ settings in (ii) achievable by density scaling in the LBM leads to better flow prediction accuracy as LBM grid is improved from coarse level to very fine level. When τ was allowed to change by not performing density scaling in (iii), the increase in grid resolution had contrary effects to solution accuracy. This highlights τ as an important parameter controlling the level of numerical diffusion at boundary condition regions seen in (iii) compared to (ii). In our main paper the τ value was fixed at 1.22.

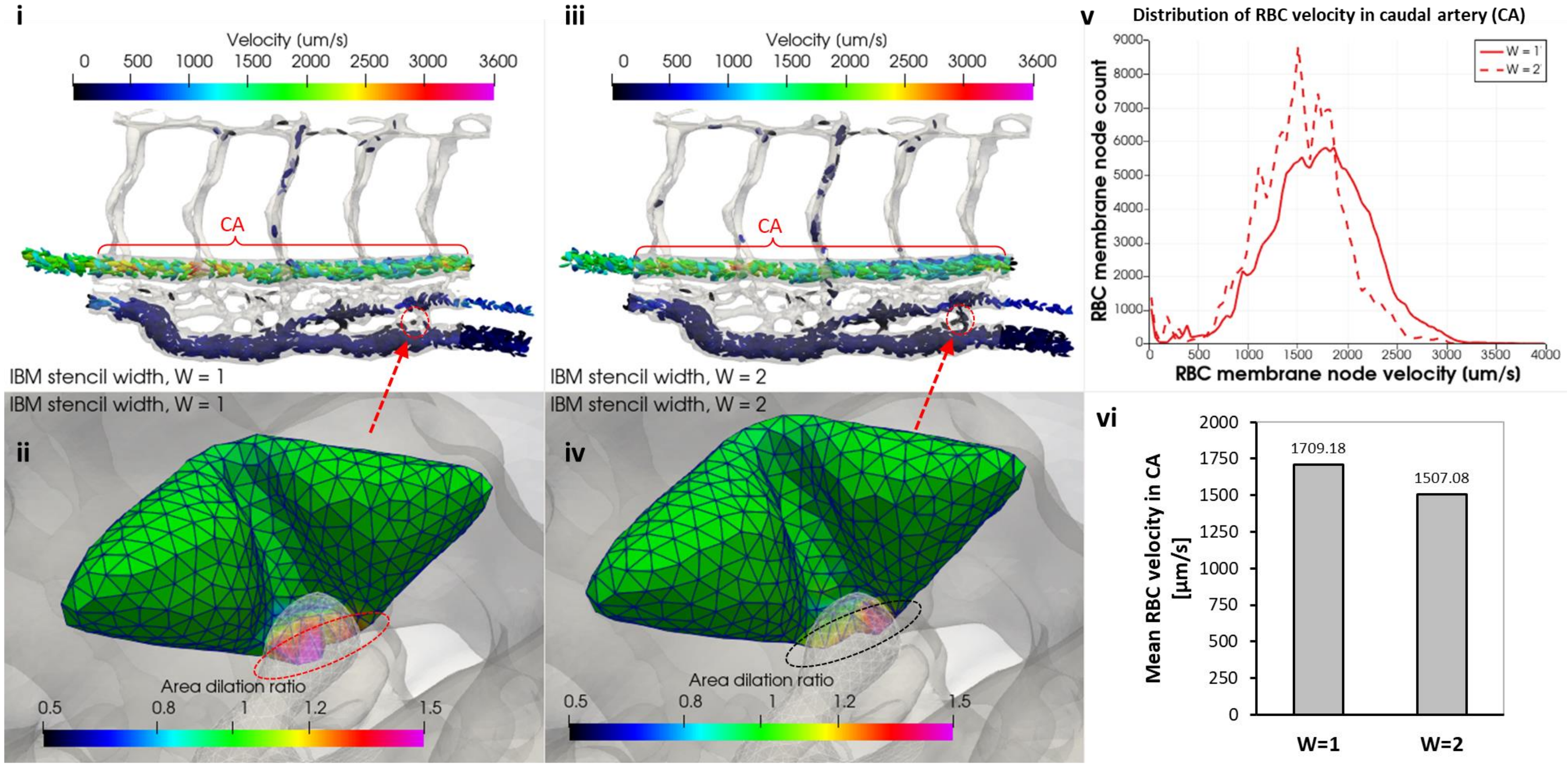

Unstable collision with protrusive wall structure: RBC is stuck artificially due to zero velocity in the stencil grid. Code eventually breaks.

Self-resolving collision with protrusive wall structure: RBC creeps away from the protrusion eventually. Code does not break

12% discrepancy in flow level under the same pressure gradients between W=1 and W=2 models

Fig J. Simulation model sensitivity to the Dirac-delta stencil width (W) employed in the Immersed Boundary Method (IBM) fluid structure interaction solver. **(i)** RBC velocity maps for the wild-type (WT) network model performed with W = 1 and **(ii)** the resulting collision instability scenario where an RBC is artificially stuck on a lumen wall structure due to zero velocity on the near-wall grid. **(iii)** RBC velocity maps for the wild-type (WT) network model performed with W = 2 and **(iv)** the resulting robust collision resolved scenario where an RBC creeps away from the lumen wall protrusion thereby avoiding extensive deformation: as the stencil is wide enough to interpolate finite velocity to the collided RBC mesh nodes, the RBC will not be subjected to prolonged stretching that can break the code. **(v)** Velocity distribution histograms for W = 1 and W = 2 models for the RBCs flowing in the caudal artery (CA). **(vi)** The mean RBC velocity in the CA for W = 1 and W =2 models, indicating a 12% higher effective viscosity in W = 2 model as a result of a more diffusive stencil.

References

1. Ye SSM, Kim JK, Carretero NT, Phng L-K. High-Throughput Imaging of Blood Flow Reveals Developmental Changes in Distribution Patterns of Hemodynamic Quantities in Developing Zebrafish. Front Physiol. 2022;13: 881929. doi:10.3389/fphys.2022.881929

2. Boal DH. Computer simulation of a model network for the erythrocyte cytoskeleton. Biophys J. 1994;67: 521–529. doi:10.1016/s0006-3495(94)80511-9

3. Pivkin IV, Karniadakis GE. Accurate Coarse-Grained Modeling of Red Blood Cells. Phys Rev Lett. 2008;101: 118105. doi:10.1103/physrevlett.101.118105

4. Fedosov DA, Caswell B, Popel AS, Karniadakis GE. Blood Flow and Cell-Free Layer in Microvessels. Microcirculation. 2010;17: 615–628. doi:10.1111/j.1549-8719.2010.00056.x

5. Evans EA, Waugh R, Melnik L. Elastic area compressibility modulus of red cell membrane. Biophys J. 1976;16: 585–595. doi:10.1016/s0006-3495(76)85713-x

6. Evans EA. [1] Structure and deformation properties of red blood cells: Concepts and quantitative methods. Methods Enzymol. 1989;173: 3–35. doi:10.1016/s0076-6879(89)73003-2

7. Rosis AD, Coreixas C. Multiphysics flow simulations using D3Q19 lattice Boltzmann methods based on central moments. Phys Fluids. 2020;32: 117101. doi:10.1063/5.0026316

8. Guo Z, Zheng C, Shi B. Discrete lattice effects on the forcing term in the lattice Boltzmann method. Phys Rev E. 2002;65: 046308. doi:10.1103/physreve.65.046308

9. Li J. Appendix: Chapman-Enskog Expansion in the Lattice Boltzmann Method. Arxiv. 2015. doi:10.48550/arxiv.1512.02599
