# Supplementary material for: A cell-and-plasma numerical model reveals hemodynamic stress and flow adaptation in zebrafish microvessels after morphological alteration
Source: PLoS Comput Biol. 2023 Dec 4;19(12):e1011665. doi: 10.1371/journal.pcbi.1011665 (PMC10721208; doi:10.1371/journal.pcbi.1011665)
Supplement: Attachment — Submitted filename: ReviewerResponse.pdf [file pcbi.1011665.s017.pdf]

Reviewer #1 (Evidence, reproducibility and clarity (Required)):

The manuscript presents a detailed numerical model of blood flow in a region of the zebrafish vasculature.

The results section is quite intense and detailed. it is difficult to understand what the authors are after. I think a rewrite would beneficial. The authors present simulations for a wild type and a couple of phenotypes. For each of these they speculate on the possible adaptation mechanism leading to the discussed phenotype, as preservation of constant wall shear stress. However, the comparison between experiments and numerical simulations is really elusive as the conclusions on those mechanisms. Overall we suggest a rewrite with clearer organisation in a way that the reader is not overflown with useless details.

We thank the reviewer for the advice on the general writing standard and data organization. We have reanalyzed experiment data and interpreted the findings more conservatively for application into the simulation models. As a result, some conclusions to the results sections have changed. Accordingly, we have done a major revision of the entire Results, Discussion and Models and Methods sections in the paper to articulate these reinterpretations while removing superfluous details that may obfuscate the data.

It is not always clear what info of the experiments are used in the simulations on top of the anatomy. Our understanding is that the pressure boundary conditions are set to match the red blood cel velocity observed in experiments. Is this always the case for the three phenotypes and which vessels ?

We thank the reviewer for the question. Only WT and Marcksl1 KO have been matched for peak velocities in the CA, CV and ISVs between experiments and simulations. WT results were compared to both the experimental reference of 27 embryos in Table 3 and also to the current experiment pool of WT (5 embryos) in Table 6. Marcksl1 KO simulation models 1, 2 and 3 were compared against the average level seen in the low and moderate perfusion Marcksl1 KO phenotypes (8 embryos) from the experiment (Table 5 and Table 6). Additionally, we also have represented the similar level of RBC hematocrit in the CA for WT model to WT experiment data from the reference cited in Table 3.

In addition to the velocity comparisons, we now use the experimentally observed trend of decreased flow rate in the CA of Marcksl1 KO experiment data to assess the model boundary conditions amongst Marcksl1 KO models 1, 2 and 3 that best reflect the experimental observations:

Page 11, lines 21 to 37

The Marcksl1 OE cannot be matched because we do not have the experiment data for that, the same goes for PlxnD1 where we have no experiment flow data. These two networks represent more conceptual discussions, particularly in PlxnD1 case where we have explicitly stated in the new discussion section:

Page 16, lines 1 to 11

There are about 7 inlets and outlets where to impose pressure boundary conditions. Can the author comment on the uniqueness of this problem?

Can different combination of pressure boundary condition leading to the same result ? In how many points/vessels is the measured velocity matched ?

We thank the reviewer on this insightful concern. Indeed, the uniqueness of flow and pressure field can be a problem without careful consideration. We have tried to address this to some extent, because CA, CV are connected by the ISV and DLAV network, to match flow velocity in all regions, the pressure distribution ought to be unique to the particular setting we employed.

As shown in table 3, average systolic peak flow velocities in the entire CA and CV encompassing the 5 ISV segment domain is matched between the simulation and the population-averaged experimental data from the experimental reference (27 fish sampled in the cited reference) for the same regions in WT network. Average systolic peak flow velocities for the 10 ISVs in the simulation were matched against WT experiment population-averaged systolic peak flow velocities in arterial and venous ISVs in the same caudal region.

Additionally, we also compared the flow velocities to the experiment conducted within this study (5 WT, and embryos). This comparison data is shown in Table 6. Admittedly the discrepancy was large for CV and ISVs regions likely due to a smaller data set sampled in this study and biological variations that happen from one experiment to another. We have acknowledged this deficiency in the revised discussion section:

Page 15, lines 3 to 9

The argument that similar beating frequency in the WT and GATA1 MO suggest pressure does not change is not clear. If the heart was a volumetric pump it would impose the same flow rate, not the same pressure. It would be more useful to measure the cardiac output in terms of flow rate in the Dorsal Aorta. Previous measurements by Vermot suggested the latter would not change much in gata1 MO. It could be that the cardiac output is the same but the vasculature network is different in a way that the shear stress remain the same. It does not look like this was checked by the authors.

We thank the reviewer for this insight. In accordance with the reviewer's suspicion, we have estimated the flow rates in the CA of *gata1* morpholino injected embryos and found the level to be similar to WT. This supports the reviewer's opinion that the heart rate similarity indicates cardiac output similarity and not arterial pressure similarity as we previously put forward. Furthermore, we have checked that the *gata1* morphants do in fact present reduced ISV diameters. In light of this reinterpretation, we performed an additional zero hematocrit model (model 3 in section 2.1). We have consequently rewritten the entire section on how RBC hematocrit modulates hemodynamics in a microvascular network:

Page 6, line 27 to page 8, line 22.

Additionaly, it would be useful to provide an effective viscosity for the different vessels, and an effective hydraulic impedance relating DP and Q to interpret the results.

We have followed the reviewer's advice and have analyzed for vessel hydraulic impedance and effective viscosity in all the network models presented. This is included in the main figures and discussion. The vessel impedances are discussed for the various models in these following parts of the manuscript:

Page 9, line 33 to page 10, line 10

Page 12, lines 3 to 7

Page 12, lines 22 to 27

Page 13, lines 8 to 12

Is the hydraulic impedance of the vessels kept constant in the smooth-geometry model? This needs clarification

The SGM diameters have been determined based on geometric averages and not impedance equivalency. The reason why we did this is because the impedance will not be known until the CFD is performed for the WT network. This is because without a pressure distribution (which cannot be determined experimentally) we cannot calculate vessel impedance since only flow can be measured and both flow and pressure are requirements to impedance calculation. Our intention with the SGM is to highlight how geometric averaging of morphological characteristics lead to incorrect flow and stress predictions. However, we understand the reviewer's sensibility and have revised the entire section of the SGM results. We have now discussed three SGM models with varying degrees of geometry simplification. The SGM1 in the revised manuscript matches WT network impedance in the ISVs by including both axial variation in lumen diameter of the WT network and the elliptical fit representation of cross-sectional skewness seen in WT ISV lumens. SGM 2 has representation of axial variation but not luminal skewness and SGM3 has only geometric average similarity to WT ISVs. The new findings and discussion can be found in the revised manuscript here:

Page 8, line 31 to page 10, line 10.

As mentioned by the authors they propose a very complex and time expensive simulation. However the results they report are kind of intuitive. Given the availability of the experimental results, would it be useful to use a simpler red blood cell model in the future, to make their simulation more practical? Or clarify when such demanding simulations can add something new?

We agree that the intuition feedback depends on the expertise of the investigator. The boundary condition selection is intuitive from the experimental findings and key data like pressures in the network cannot be measured. Furthermore, population-averaged flow data does not always match the flow-to-geometry situations that vary from sample to sample, thus demonstrated by the high margin of prediction discrepancy for flow velocities in table 6. We have discussed these challenges and our recommendations for improvement in the Discussion section:

Page 15, lines 22 to 33

Page 16, lines 12 to 15

Page 16, lines 20 to 27

On the topic of RBC model simplification, we agree with the reviewer that our work suggests the methodology would benefit from a further coarse-graining approach to the RBC phase. Accordingly, we discussed the possibility of using a low-dimensional RBC model already published in literature:

Page 15, lines 22 to 33

The authors should check their references as this is not the first time work has been done on the topic. Would be good to have a check in the work of Freund JB and colleagues, as well as Dickinson and colleagues and Franco and colleagues to discuss how the work compares. There may be interesting work in modelling cardiac flow forces in the embryo too.

The authors should check their references as this is not the first time work has been done on the topic. Would be good to have a check in the work of Freund JB and colleagues, as well as Dickinson and colleagues and Franco and colleagues to discuss how the work compares. There may be interesting work in modelling cardiac flow forces in the embryo too.

Thank you for referring us to other publications that are related to our study. To our knowledge and after performing publication search on these authors, we find that although Dickinson and colleagues performed experiments to examine the effects of perturbed blood flow on vessel remodelling (Udan et al., 2013), they did not perform any numerical modelling to calculate hemodynamic forces such as WSS and luminal pressure. Instead, changes in vessel morphogenetic process were only correlated with blood flow velocity. In our study, we attempt to quantitatively correlate WSS and pressure distributions within a vascular network. Franco and colleagues (Bernabeu et al., 2014) developed PoINet to model haemodynamic forces in mouse retina model of angiogenesis. From what we understand, PoINet is different from our 3D CFD model by 1) not having red blood cells incorporated in their model and as such, the blood viscosity prediction is modelled using shear-rate dependent formulation and not through red blood cell hematocrit, 2) cross sections of blood vessels are assumed to be circular and

therefore have no irregularity and 3) live imaging of blood flow is difficult in mouse retina therefore preventing accurate boundary conditions for the model.

We have included the reference to work of Franco and colleagues:

Page 15, line 6 to line 9

Page 9, lines 23 to 27

Freund JB indeed has had extensive work on RBC and cellular flow in microvessels. We have included a reference of his work in:

Page 14, line 38 to page 15, line 3

Reviewer #1 (Significance (Required)):

The authors discuss the applicability of a detailed numerical model of blood flow in a region of the zebrafish vasculature.

We are not expert in the lattice boltzmann method used here, but the results are what it would be expected from a physical stand point, and together with the information from the method section, we do not have major concerns about the numerics.

Reviewer #2 (Evidence, reproducibility and clarity (Required)):

Summary: The authors report corroborating numerical-experimental studies on the relationship between morphological alterations (e.g. vessel lumen dilation/constriction, network mispatterning) and hemodynamical changes (e.g. variation in flow rate, pressure, wall shear stress) in the vascular network of zebrafish trunk circulation. Various physiological or pathological adaptation scenarios were proposed and tested, with a range of simulation and experiment models. Where I found it a solid piece of work supported by abundant data, certain aspects need to be clarified/enhanced to improve the scientific rigor and potential impact of the manuscript. Below are my detailed comments in the hope of helping the authors improve the manuscript's quality.

Major comments:

1. Cellular blood flow in vascular networks has been extensively studied in recent years by existing computational models (some of which were published open-source) with similar methods and features to the one proposed by the present work. Can the authors be more explicit about the original contributions of the current model, and provide evidence accordingly (e.g. Github repository or code resources)

The RBC model is essentially the model developed by Fedosov and colleagues (Fedosov, et al., 2010). Likewise, the LBM solver for fluid flow calculation is not novel. Following the reviewer's advice, we have removed the details of these non-novel aspects of the methodology and placed them in sections E and F of supplementary material instead. The new Models and methods now show condensed descriptions of the three numerical solvers used, more details into how cellular contact and collisions are handled, and a new addition of a grid independence matrix discussion section:

Page 17, line 34 to page 22, line 21.

With regards to evidence of our contribution in the form of a publicly available code, we have uploaded our numerical solvers code ZebraflowWT.c on Github along with the dependencies required to perform a simulation run:

https://github.com/SweSoe/Zebraflow/tree/0.1.0

The repository version used in this study has also been indexed with Zenodo:

https://doi.org/10.5281/zenodo.7960906

2. Crucial details for the simulation setup and model configuration are missing. What were the exact boundary conditions (e.g. inlet and outlet pressures) and initial conditions (e.g. feeding hematocrit of RBCs), and how the numerical-experimental validation process of "to match the velocities of various segments of the network by iteratively altering the pressure inputs ..." as stated on page 13 (lines 1-2) was performed for simulations in this work?

We apologize for the vagueness of our description on how numerical to experimental validations were performed. As replied to reviewer 1 for a similar clarification, we have indicated in Table 3 how average systolic peak flow velocities in the entire CA and CV encompassing the 5 ISV segment domain were matched between the simulation and the population-averaged experimental data for the same regions in WT network. Average systolic peak flow velocities for the 10 ISVs in the simulation were matched against WT experiment population-averaged systolic peak flow velocities in arterial and venous ISVs in the same caudal region.

With regards to what iterative alterations of pressure inputs mean, we monitored the average systolic peak velocities and hematocrit levels in CA, CV and ISVs in intervals of 5 cardiac cycle intervals before manually correcting the pressure input levels to better match average systolic peak velocities in these vessels from the experiment averages. Since we are using population averaged flow data, we do not expect their levels to match the levels in a particular fish-specific geometry, the degree of discrepancy between experiment averages and the model predictions of systolic velocities can be large (Table 6). Admittedly, this is one of the weaknesses of our approach and this limitation is stated in the Discussion section:.

Page 15, lines 22 to 26

As RBC flow typically requires roughly 5 cardiac cycles of flow to reach flow development this process of iterative correction typically takes place over 10 to 20 cardiac cycles. We understand that validation may be a subject of keen interest to readers, hence we have now briefly described the solution initialization and flow development protocol in our modeling approach here:

Page 5, lines 7 to 13

What lattice resolution was used for the flow solver and was the RBC membrane mesh chosen accordingly? Were there any sensitivity analysis (regarding pressure input) or grid-independence study (regarding lattice resolution)

We originally decided on the grid (ΔX) and time (ΔT) discretization resolutions (0.5 µm and 0.5 µs) based on the acceptable computing turnaround time for each model within our scale of resources. We have now included a section on the grid independence matrix in Models and Methods:

Page 21, line 4 to page22, line 21

3. Details of the statistical tests (type of tests used, assessment of data normality, sample size etc.) should be given in the figure caption where applicable (e.g. Fig. 3C, Figs. 7-9). We apologize for the lack of clarity. All statistical tests used have now been mentioned at least once in each section of results and also in Figure captions wherever significance bars are displayed.

The regression models should also be used with caution, e.g. in Fig. 4B, why should data from two different fish types, namely Gata1 MO and WT, be grouped to fit a linear regression model?

We understand the reviewer's concern that two population data sets should not be carelessly pooled together for regression analysis without adequate justification. In this case we are utilizing *gata1* morpholino injection as a means to alter hematocrit level. There is no reported side-effect as to the best of our knowledge, only hematocrit and possibly hemodynamics and morphological response related to hematocrit level should be affected. Moreover, we have mislabelled the companion set to the *gata1* morpholino as WT, the data is in fact data from control morphants and not WT. This change has been applied to Fig. 3 graphs and Table 4 and results section:

Page 7, lines 16 to 31

Finally, as we want to generate a continuum range of varying hematocrit for embryos of the same developmental age. In this regard, we think that within the scope of our intentions and well-accepted usage of *gata1* morpholino as a hematocrit reduction protocol it is reasonable to pool the two data sets together for regression analysis.

4.I found the data presented in Fig. 7 insufficient to confidently exclude the numerical models 2, 3 but favor model 1 as the adaptation scenario for the Marcksl1KO case. The first question is, how are the threshold RBC perfusion levels determined to categorize the experimented Marcksl1KO fishes into four groups, i.e. "high", "moderate", "low", "zero"? The authors also need to justify why the "high", "moderate", "low" groups can be mapped to the three modelling scenarios (namely models 1, 2, 3) is it just because "a qualitative match with the experimental trend of ascending CA blood velocity" (Fig. 7F)?

We thank the reviewer for his interpretation of our results. Firstly, we apologize for generating the confusion but we are not trying to map simulation models 1, 2 and 3 to high moderate and low groups respectively in Fig. 7. The high, moderate and low categorizations of experimental Marcksl1 KO phenotypes are based on RBC flux levels observed experimentally. We are trying to ascertain which Marcksl KO phenotype the models 1, 2 and 3 fit, if they do fit the experiment trend at all.

Second, in Fig. 7C, it is shown that no significant difference exists between the "high" group and the WT in their average ISV diameter, then what is defining that group as Marcksl1KO type ?

We apologize for the confusion generated. High flow phenotype is similar to WT flow, the diameter is also similar to WT. In Marcksl1 KO mutants we don't always see clear phenotyping and often a range is presented from mutant to mutant. Hence the high group is essentially morphometrically and hemodynamically similar to WT, the only reason we know it is a mutant because we have genotyped the zebrafish (*marcksl1a$^{-/-}$;marcksl1b1$^{-/-}$*).

Third, a central assumption here is using heart rate as a measure of the pressure drop in different fish individuals (Fig. 7D). Can't two fishes with similar heart rate have distinct pressure drops in the trunk due to difference in network architecture and topology, vice versa?

We agree with the reviewer's opinion and now feel that our initial proposition was naïve. After addressing the interpretation of heart rate similarity in the *gata1* morphants with more convincing CA flow rate estimations, we now believe that heart rates might not be useful indicators of flow or pressure levels in the network. Instead, cardiac output in the form of CA flow rate as reviewer 1 has suggested might be a better indicator. As the reanalysis has dismantled the earlier interpretation, and found that based on the flow

rate estimation for the CA, Marcksl1 KO networks have reduced blood flow rates in the CA.

Page 11, lines 21 to 25

This finding has been incorporated into the consideration of flow adaptation scenarios predicted by the simulation models accordingly in the revised manuscript:

Page 11, line 25 to page 13, line 25

Fourth, the authors should explain why a power-law fit (note that it is not "exponential" as stated on page 10, line 3) should be adopted for the regression analysis in Figs. 7E-v,vi (a useful reference may be Joseph et al. eLife 2019: 10.7554/eLife.45077).

We thank the reviewer for the useful reference and the careless mislabeling of regression curve used. This figure has been redone and a linear regression is instead used that does not attempt to imply any physical law for a power or exponential fitting.

Change made: Fig. 7C

Minor comments:

1. The state of art of cell-resolved blood flow models employed to simulate microcirculatory hemodynamics is not accurately described in the introduction (page 4). More recent works should be cited and critically reviewed to present a fair view on the novelty of the computational model developed herein.

We apologize that the models were mentioned in a passing manner. However ,the need for brevity in introduction somewhat limits their expansion. We have instead gave more direct discussion on similar studies and their relevance to our present work in the Discussion section:

Page 14, line 9 to page 15, line 17

2. It is unclear what "realistic representation of local topologies in the network" (page 7,

lines 28-31) means as a claim of novelty. If it means vessel "diameter variation", this geometric feature has been modeled by the works the author referenced (namely Roustaei et al. 2022, Zhou et al. 2021). If it means something else, for example, unsmooth or non-circular vessel surface (or "irregularity of the local endothelium surface" as mentioned on page 5, line 2), then strangely the effects of such features are actually not described in the manuscript.

We apologize for not meeting the expectation of novelty as claimed. We see value in the SGM study matrix have now generated data on three SGM scenarios. The SGM1 in the revised manuscript matches WT network impedance in the ISVs by including both axial variation in lumen diameter of the WT network and the elliptical fit representation of cross-sectional skewness seen in WT ISV lumens. SGM 2 has representation of axial variation but not luminal skewness and SGM3 has only geometric average similarity to WT ISVs. Essentially the comparison between SGM1 and SGM2 highlights the role of luminal cross-sectional shape skewness while SGM2 to SGM3 highlights the role of axial variation in luminal diameter. With this new SGM data set, we think we can better qualify the aspiration of demonstrating how vessel shape “irregularities” can alter network hemodynamics. The new findings and discussion can be found in the revised manuscript here:

Page 8, line 31 to page 10, line 10.

3. Why should Fig. 8 contain data from Marcksl1KO model 2? The scenario underlying model 2 was rejected earlier in the manuscript (see point 6 above), and the Marcksl1KO model 2 data are not mentioned in the text when describing the results of Fig. 8, either.

We have reanalyzed the experiment trend and rewritten the outcome of this results section. In summary, both models 1 and model 2 meet the trend of flow rate reduction (with respect to WT levels) in the CA observed in the experiment. Hence, model 2 inclusion is relevant to the WSS analysis. The changes pertaining to this can be found here:

Page 11, line 21 to page 13, line 25.

4. It is a dense article with loads of data, which is an advantage but only if appropriately streamlined. More subheadings should be considered, especially for section 2.3 (for

which the current subsections appear mistaken, 2.3.1 followed by 2.4.2) The manuscript could also benefit from restructuring through optimal combination of simulation visualizations and quantitative analyses. For example, in Fig. 6, not all simulation snapshots are needed here (it is difficult to visually compare the changes between different cases), whereas some quantification in the form of histograms or boxplots will be handy for the readers to note the variation of WSS magnitudes and ranges.

Thank you for the advice, we removed the unnecessary graphical plots and refer to simulation videos in supplementary data instead for such cases. The bad indexing of results subsections has been fixed, while new subsections have been made for better directional narrative to the paper. These changes are colored in red throughout the revised results section:

Page 4, line 37 to page 14 line 16

5. Related to point 8, the authors could also consider integrating or synthesizing the analyses for individual aISVs and vISVs presented in various figures. Current descriptions for the ISV data appear scattered with frequent exceptions to the summarized trends or relationships. Some minor formatting issues should also be addressed, e.g. the confusing color codes in Figs. 9D-i, E-i.

Thank you for the advice, we have now pooled aISVs together into one group and vISVs into another, instead of discussing data trends on each of the 10 ISVs.

6. The mispattening case presented in the end of the results section (section "2.4.2") is interesting but appears loosely connected to the preceding contents. Also, it seems not even mentioned in the discussion section.

We agree that the mispatterning case has been only tangentially relevant to the rest of the manuscript. We have linked the topic thematically by network alterations transforming network flows. It is also now included in the discussion section here:

Page 16, lines 7 to 11

7. Finally, apart from the effect of topological features on local blood flow, the authors

should consider the global flow redistribution arising from the network structure (useful refs. Include Chang et al. PLOS Computational Biology 2017: 10.1371/journal.pcbi.1005892; Meigel et al. Physical Review Letters 2019: 10.1103/PhysRevLett.123.228103; Schmid et al. eLife 2021: 10.7554/eLife.60208).

Thank you for the additional references. These are solid pieces of work that have been added to the discussion here:

Page 16, lines 21 to 27

Referees cross-commenting

This review report resonates with mine from an experimental perspective and I agree with all points made regarding issues of the current manuscript that the authors need to address with a revised version.

Reviewer #2 (Significance (Required)):

Significance: The particular merit of the work lies in its comprehensiveness of design and abundance of data, which will be of great interest to both the computational and experimental communities in this research field. However, some crucial details (especially with respect to the modelling aspects) are missing, thus hampering the scientific rigor and potential impact of the work. Furthermore, certain justifying statements appear speculative and inconclusive to explain the obtained data, especially regarding the effect of boundary conditions and systemic parameters. The citation of references (some not cited, some cited already but not properly discussed) also needs to be enhanced with engaging discussions to better bridge the findings of the current work (e.g. RBC partitioning in vascular network, effect of WSS on vasculature morphogenesis) with recent works on this research topic.

References

Fedosov DA, Caswell B, Karniadakis GE. 2010. A Multiscale Red Blood Cell Model with Accurate Mechanics, Rheology, and Dynamics. *Biophys J* 98:2215–2225. doi:10.1016/j.bpj.2010.02.002

Freund JB, Goetz JG, Hill KL, Vermot J. 2012. Fluid flows and forces in development: functions, features and biophysical principles. *Dev Camb Engl* 139:1229–45. doi:10.1242/dev.073593
