# Supplementary material for: A cell-and-plasma numerical model reveals hemodynamic stress and flow adaptation in zebrafish microvessels after morphological alteration
Source: PLoS Comput Biol. 2023 Dec 4;19(12):e1011665. doi: 10.1371/journal.pcbi.1011665 (PMC10721208; doi:10.1371/journal.pcbi.1011665)
Supplement: Attachment — Submitted filename: 2ndRevResponsOctober12th.docx [file pcbi.1011665.s018.docx]

We thank both reviewers for their insightful comments and earnest concerns over deficiencies in our communication of results and discussion. We have amended the Results, Discussion and Methods sections in the revised manuscript to address the concerns from the two reviewers. Manuscript headings have also been revised to remove header numbering and instead follow PLOS Computational Biology’s 3-level heading formats. Our response letter is coded in color for convenient reading. The reviewer’s original comments are coded in black, our response and explanations are in blue. Quotes of the previous manuscript version are given in green. Quotes of text from the new revised version of the manuscript is coded in red.

Changes made between the previous manuscript and the revised manuscript have been annotated in red font for the text in the revised manuscript.

Reviewer #1: The authors have carefully addressed the reviewers’ comments (including my own) with additional simulations and benchmark tests, and I find the quality of the paper largely improved and scientifically sound. However, the minor issues below should be addressed before its publication.

1. The revised manuscript is very dense with overflown data & analysis, and difficult to navigate. One reason behind this is the evenly detailed/weighted description of less important data (e.g. exhaustively listed percentages which are not needed everywhere or statistically insignificant values).

We apologize for the excessive usage of percentage metrics and indiscreet mentioning of statistical significance parameters. We have revised the results section to remove the indiscriminate usage of such metrics and limited them only for important data quantifications that we wish to highlight. All statistical testing information has been moved to figure legends and removed from the main text.

Another reason is the frequent jumping between experiment and simulation data, sometimes without clear signposting. The authors may consider adding a signposting paragraph at the beginning of the results section to offer a point of entry before the readers dive in the comprehensive results sections.

We thank the reviewer for the advice. We have signposted the beginning of paragraphs when we switch the narrative from experiment to simulation or vice versa. We hope that the segue between the two methods is smoother and more coherent connections are apparent to the reading audience now.

2. Related to point 1, avoid confusing classification of cases, e.g. “Model 1, Model 2, Model 3” for the WT in section 2.1 and “model 1, model 2, model 3” again for the Marcksl1 KO in section 2.2 (consider distinguishing them in both the figures and text). Some supplementary movies may be combined, e.g. velocity, pressure and WSS videos for the same case.

We apologize for the confusing classification. We now refer to the WT model simply and consistently as WT in all the sections. The two zero hematocrit simulation models are now referred to as NoRBC1 and NoRBC2. Likewise, in the Marcksl1 KO cases we refer to the 3 KO models as ML1KO1, ML1KO2 and ML1KO3. The model classification nomenclature are now simply enumerated abbreviations with clear connection to the phenotype classification, we hope this avoids confusion in our updated manuscript. Movies have also been combined to include velocity, WSS and pressure animations into a single video for each network phenotype.

3. Not sure the individual section 2.3.2 for pressure redistribution is necessary, which is linearly correlated with WSS for designated vessel segments.

The section has been combined with the next section in accordance with the reviewer’s advice.

4. Section 2.3.3, last paragraph: “based on vessel impedance estimated by vessel size”. This contrasts with the definition of vessel impedance on Page 9.

We apologize for the confusion generated; the contradiction was not what we meant. We were trying to point out the opposite, that simply assuming inverse relationship between size and WSS magnitude was in fact the wrong understanding of flow impedance. We have amended this statement to avoid inviting this unintended misinterpretation.

The original statement on Page 13, Lines 17 to 19 of the previous manuscript read:

“Without a CFD analysis it would be difficult to predict the outcome just based on vessel impedance estimations by vessel size.”

The amended statement now on Page 13, Lines 36 to 38 reads:

“Without a CFD analysis it would be difficult to predict the outcome just based on simple assumptions that vessel diameter reductions always lead to local WSS increase or vice-versa.”

5. In the discussion section (e.g. page 16, paragraph 1), the authors are suggested to link findings in this study with prior studies, either supporting or contradicting current understanding of how mechanical cues shape vascular development (as nicely discussed in the introduction section). A missing opportunity is to bridge the findings of vascular morphology-triggered flow adaptation in this study with potential WSS heterogeneity-triggered vascular remodelling as reported by previous studies.

We apologize for the lack of substantial linkage of our results to previously published studies that are of similar themes and methods to our work in the previous manuscript version. We have re-written most of the discussion section to address this deficiency:

Page 15 Line 3 to Page 19 Line 8

With specific regards to vascular remodeling outcomes due to WSS heterogeneity in a network, we have expanded this discussion in the revised manuscript for an overview on flow and WSS effects on plexus remodeling from prior studies:

Page 17, Lines 8 to 24:

“Since WSS is known to promote vascular endothelial growth factor (VEGF) expression in ECs [60], persistent dead-flow and low WSS regions in a lumen network are expected to receive dysfunctional remodeling cues. It is known from other studies that flow and WSS participate in plexus remodeling. In a study examining DLAV remodeling, circulatory flow and VEGF expression was found to promote initial plexus expansion between 32 – 48 hpf and by 72 hpf promote DLAV fusion into a continuous vessel [12]. Abrogation of both flow and VEGF signaling caused regression in the DLAV remodeling. In the CVP, low WSS regions correlated with sites for intussusception whereby existing lumen split to form new plexus branches and optimize network WSS to more homogeneous distribution [10]. In avian capillary plexus remodeling, vessel sprouting occurs in low WSS locations from vessels of lower pressure towards vessels with higher pressure, often avoiding flow-convergent bifurcations [61]. In the case of mouse retina vascular plexus (postnatal day 5), local regions of stenosis and vascular regression correlated with regions of low RBC perfusion [28]. Unfortunately, the experimental studies with PlxnD1 morphants [45] and *out of bounds* (OBD) mutants [44] do not discuss the subsequent remodeling outcome of these mispatterned ISV networks. However, we can speculate from these other plexus remodeling studies that the heterogenous WSS patterning is likely to result in regression or stenosis in the low WSS dorsal ISV-DLAV connections bypassed by the shunts.

Also, the RBC flow studied here should be compared with similar studies, such as the cited work (Zhou et al., 2021), who observed fairly dynamical RBC perfusion in the CVP of zebrafish vasculature and provided some insights on the role of RBCs in remodelling the vascular network.

Proper linkage of the work by Zhou et al., 2021 with regards to our work with gata1 MO experiments have now been undertaken in the revised manuscript. Specific discussion of this cited reference can be found here:

Page 15 Lines 21 to 30:

“Indeed, RBCs can be integral to vascular remodeling as demonstrated by Zhou and colleagues [28] where a significantly decreased rate of CVP width reduction in *gata1* MO zebrafish was observed in comparison to control MO between 50 to 72 hpf. This implied a delayed CVP remodeling for *gata1* MO zebrafish. Our own experiments with gata1 MO versus control MO zebrafish indicated a morphological adaptation of reduced ISV lumen diameters in the *gata1* MO group at 53 to 57 hpf. Since ISV lumen diameters have been reported to increase in tandem with rises in CA and CV hematocrit from 48 to 72 hpf [17], the ISV diameter reduction in *gata1* MO zebrafish in our study indicates a similar delay in the remodeling stage for ISVs in hematocrit-reduced zebrafish networks. Hence, our study contributes to the growing understanding that RBCs in zebrafish trunk network flow appear to participate in the remodeling of network vessels.”

6. Why adopt pressure boundary conditions at the CA/CV inlets/outlets? Isn’t it easier to directly prescribe inflow/outflow BCs given these are experimentally measured already? This way, designated discharged haematocrits can also be easily fed at the inlets for given flow rates.

Thank you for the question, we think there is a misunderstanding of what we can measure from the experiments, possibly due to insufficient clarification in our previous manuscript version. We cannot use inflow/outflow BCs because our RBC-tracking measurements can only measure the highest velocity in the lumen which by definition is the centerline velocity. Since vessel lumens are 10 to 30 µm in diameter in the trunk network, fairly large RBCs that are also around 10 µm in their longest axis make bad particle tracers for constructing radial velocity distributions from imaging experiments. There is also no good analytical velocity distribution function based on centerline velocity alone, considering that we do not know the degree of bluntness in the flow velocity radial profile. Furthermore, the lumen cross-sections are not perfectly circular. Hence, we have decided to utilize pressure BCs to circumvent this difficulty.

The hematocrit designation is a separate challenge independent of the choice between flow or pressure BCs. Even if flow BCs are utilized, the control of domain hematocrit still follows the same procedure of staging the correct RBC number density in the reservoir domains. The use of pressure BCs neither helps nor hinders this process. The populating of RBCs within the network domain to desired hematocrit can be sped up by initializing the flow with initial seed concentration of RBCs, we have tried this method to mixed success. This approach is not reliable because it is difficult to configure undeformed RBCs above 10% hematocrit into the irregular shaped network domain because RBCs typically have to deform to fit into the lumen spaces, especially at narrower regions. It is impossible to place RBCs in ISVs without any deformation. We cannot arbitrarily prescribe RBC deformations just to enable the desired hematocrit quickly (we also tried this with the result of frequent excessive unphysical deformation and degeneration of RBC mesh), it has to follow the momentum balances prescribed by laws invoked in our model. Hence it is a more stable and robust simulation process to feed RBCs gradually into an initially empty network using the reservoir domains.

We think the reviewer’s clarifying question is important and may be something that requires mentioning in the paper. As such, we have added more details on the BCs and some comments about why we cannot use flow BCs instead of pressure BCs:

Page 5, Lines 22 to 33:

“Notably, we could not apply flow inlet BCs based on the experimental measurements. This was because the full 2-D distributions of the cross-sectional velocity required for flow-input BCs at the anterior and posterior grid points in the LBM model is not measurable by RBC-tracking in our experiments which only provided the lumen centerline velocities. Critically, the relatively large size of RBCs compared to the lumen diameters prevented particle tracking velocimetry from building radial distributions of flow velocities in a lumen. Furthermore, while the cross-sectional velocity distribution is known to adopt a blunt (plug-like) flow profile around the lumen centerline velocity, non-circular lumen cross-sections in the network geometry make it difficult to define analytical velocity distributions as functions of the lumen radius. We also cannot determine the degree of bluntness a priori as this itself is the subject of evaluation by CFD which assess the rheological dynamics of cellular interactions in the blood mixture (discussed in the next subsection).”

We think part of the expectation for using flow inlet/outlet BCs may stem from the fact that we reported CA flow rates for the gata1 MO experiments and Marcksl1 KO experiments. This was requested by the reviewer in the previous round of critique and originally outside of our experiment design. Typically, radial distribution of flow velocities in the lumen are required in order to calculate the flow rate which is an areal integration of the lumen velocity distribution. We would like to clarify that we do not have the velocity distributions in the lumen cross-sections. We circumvented this by estimating the flow rate instead based on the bluntness relation between bulk flow velocity and centerline velocity. Typically, in Poiseuille flow, the bulk flow velocity is 0.5 of the centerline velocity. In blood and other non-Newtonian flows the ratio will be between 0.5 and less than 1; the more blunt the profile is, the closer the ratio is to 1. Based on the CFD result of 20% hematocrit flow (no RBC aggregation) in a 30 µm diameter lumen in section 2.1, we found the ratio to be 0.64. Relating this level to the hematocrits observed in experiments we made linear estimates of the ratios and bulk flow velocities in each sample in order to estimate their flow rates. In order to avoid mischaracterizing what we actually measured from flow imaging, we have provided details of how flow rates were estimated in the revised supplementary materials document:

Supplementary S2 Text, Section A

7. The authors should briefly justify why nucleated RBCs are modelled in relation to the 2 dpf zebrafish studied (also, does the RBC size match?).

Unlike mammalian RBCs which have nuclei only at the reticulocyte stage, zebrafish RBCs are nucleated even after maturing. Despite the lack of data on their exact properties, we have included the nucleus in modelling the RBCs because they are physiologically present. For the benefit of audience who might not be familiar with the differences between zebrafish RBCs and human RBCs, we have made a clarifying comment with appropriate citation that nuclei are present in zebrafish RBCs:

Page 20, Lines 23 to 26:

“A major difference between zebrafish RBCs and human RBCs apart from the shape, is the presence of nuclei even in mature RBCs for zebrafish [4,70]. Our model therefore includes the nucleus as a three-dimensional meshwork where enjoining edges act as compressible springs for the prescription of nuclear deformation.“

It may be beneficial to parametrically explore their contribution to RBC mechanics but we have decided to keep that outside of our paper’s present scope. We wish to have a separate study on parametric contributions of different RBC properties on zebrafish blood flow once we have decided on the experimental means of manipulating such parameters to complement the discussion of CFD predictions.

With regards to RBC size, we have followed the literature for the resting dimensions of zebrafish RBCs observed in blood smear tests. The reference dimensions are now included in the amended manuscript:

Page 20, Lines 19 to 21:

“The zebrafish RBC is modelled as a flattened ellipsoid capsule (10 by 7 µm in the long and short disc axes, respectively and 3 µm in thickness [67]) with an RBC membrane shell enveloping a cytosolic interior and a nucleus (Fig. 1C).”

Note a potential conflict of zebrafish age, 53-57 hpf on page 7.

We apologize for not understanding exactly what conflict the reviewer is referring to. We assume that the concern is over the 4-hour time range between the gata1 MO and control MO samples. This arises from the way we conduct our experiments where we keep the zebrafish alive during the experiments. As such, the embryos continue to develop as we image embryos sequentially. As Gata1 MO and control MO embryos were all imaged on the same day, with the control MO samples imaged after the gata1 MO samples, the control morphants are a few hours older than the gata1 morphants. We do not think that the 4-hour time difference will make a significant difference to the experimental data obtained and is a conflict.

8. In addition to Hemocell (Zavodszky et al, 2017), other widely used cell-plasma LBM flow solvers should also be mentioned, such as Hemelb (Zhou et al., 2021), Palabos (Latt et al., 2020: 10.1016/j.jocs.2020.101153) and Harvey (Ames et al., 2020: 10.1016/j.camwa.2020.03.022)

We thank the reviewer for his/her extensive coverage of other cell and plasma CFD solvers and studies. We have included them in our discussion.

Page 15, Lines 7 to 17:

“The CFD model described in this work is not the first to implement cell and plasma flow models in examining microvascular flows. HARVEY [46], Palabos [47] and HemeLB [48] are Lattice Boltzmann Method (LBM) fluid transport solvers like our model. They have been coupled to cellular deformation models such as Hemocell [49] to model RBC deformability in cell-force spectroscopy tests [50]; WSS augmentation in capillary ECs by circulating tumor cell interactions [51]; diabetic RBCs in microaneurysm flows in retinal microvessels [52]; cellular margination [53], hematocrit partitioning [54], and phase-separation in RBC-rich microvascular bifurcations [28]. Likewise, other CFD solvers have studied RBC deformation and interactions with capillary walls [55], cell-free layer recovery post flow disturbances [34,56] with implications of these effects to flow and vascular pathophysiology [32]. The common impetus in all of these studies is that the RBC phase is directly responsible for the flow physiology being studied.”

Reviewer #2: The manuscript presents a 3-D+time Computational Fluid Dynamics (CFD) model depicting microcirculation in embryonic zebrafish tail, which incorporates a blood transport model of cells and plasma and offers novel insights into microvascular circulation, remodeling, and adaptation. To further strengthen the paper and its relevance to the intended readership, the following points are suggested for revision:

Major comments:

1. A noticeable gap in the manuscript pertains to the image segmentation and analysis needed to construct the 3-D computational domain. The process of image segmentation, necessary for the reconstruction of the vessel network, is currently unclear. Further explanation and details regarding the segmentation algorithm would be beneficial. The usage of Paraview for this purpose seems unorthodox; could you confirm if a thresholding algorithm was applied given the apparent non-smoothness in the computational domain (Fig.1 B – ISVs at left)?

We thank the reviewer for the critique and advice. While we cannot comment on orthodoxy, Paraview provides a very effective 3D interaction environment for voxel segmentation and lumen wall surface mesh generation from binarized rectilinear 3D image grids. The thresholds applied are manually set to the custom level in different regions of the trunk network. Aiding in this process is the use of both endothelial cell (EC) cytosol marker (channel 1) and lumen marker fluorescent dextran (channel 2). The threshold level for channel 2 is selected based on the regional intensity level that clips at the boundaries where channel 1 and channel 2 voxels colocalize. As the EC marker is not a continuous labelling with gaps in signal in some regions, it is not possible to automate the colocalization algorithm. The procedure is briefly described now in the amended methods section and for greater clarity Movie12 has been uploaded that provides a walkthrough guide on steps and procedures involved in the image segmentation and mesh generation from the fluorescence confocal microscopy images:

Page 19 Line 31 to Page 20 Line 10:

“3D reconstruction of lumen morphology and network was performed using Paraview (https://www.paraview.org/) to obtain an in-silico model of the zebrafish trunk network (S12 Movie). The 3D tif image was imported into Paraview and using the in-built software filters of “clip” and “threshold”, the lumen geometry was segmented in a regionally dependent thresholding of the dextran tetramethyl rhodamine channel (channel 2). The regional customization of clipping threshold intensity was manually determined within the Paraview graphical user interface (GUI) where the EC cytosol marker channel (channel 1) was used to identify the appropriate channel 2 threshold intensities based on the boundaries where channel 1 and channel 2 voxels co-localize. This requires a graphical processing environment where 3D interrogation and manipulation of voxel data was user-friendly, which Paraview’s GUI provides. Finally, the surface mesh of the lumen wall was obtained using the “Isosurface” filter (also known as “Contour” on some versions) in Paraview. For details of this procedure please refer to S12 Movie.”

2. The flow patterns, notably in ISVs, are heavily influenced by the structure and connectivity of the DLAV. The addition of a discussion that evaluates the reliability of using the DLAV structure and connectivity pattern from a particular section of one zebrafish tail, and whether these findings can be generalized, would potentially support the argument and overall conclusions. The model, as it stands, presumes a paired aISV and vISV at each branching point along the CA, which is not always the case. This observation extends to the application of changes in vessel diameter in the Marksl1 models.

The reviewer points out an important concern with regards to DLAV and ISV structural remodeling. We wholeheartedly agree with the reviewer but we think this topic is better explored on a larger network model, possibly a model of the entire zebrafish trunk network. We are overburdened by the scope and we would like to pursue this topic for a new study instead where methodology elements of our current work have undergone further improvement. Nevertheless, we included some commentary of this topic in our discussion, acknowledging this omission as a limitation in our study’s scope. This discussion can be found in:

Page 18, Lines 17 to 30:

“The final limitation is that we did not discussed variations in DLAV structure and the aISV-vISV distribution pattern in the network due to the overexpansive scope of such a study. The WT CFD model reconstructed from imaging has an ISV patterning where all contralateral ISV neighbors were arterio-venous (A-V) pairings. Of the 8 ipsilateral pairings, 4 were A-V, 2 were A-A and 2 were V-V. This corresponds with findings by Geudens and colleagues who reported frequent A-V ISV pairings, especially contralaterally [7]. Since our CFD network phenotypes were direct modifications of the WT network, all phenotypes followed the same ISV pair patterning. We did not study if there were shifts in this pairing bias amongst the morphologically and hemorheologically altered zebrafish. Likewise, different DLAV architecture was not investigated as this has not been reported in Marcksl1 mutants [43] nor observed in our imaging experiments. However, on an entire trunk network scale, there may be interesting cases of local DLAV variations amongst zebrafish that is related to the pairing pattern for ISVs. In addition to the work by Geudens et al. [7], the analyses of ISV patterning and DLAV remodeling with respect to WSS patterning in the network would be enlightening. We intend to study these effects more thoroughly in a separate study.”

The manuscript does not provide clarity on the specific section of the zebrafish tail (anterior or posterior) considered for the experimental diameter measurements. maybe the marcksl1 expt images – show the CA and CVP

We apologize for the lack of clarity on where flow and diameter measurements were taken. This is now provided for in supplementary figures S1D for gata1 MO, S1H for control MO experiments, S2D for WT experiments and S3D, S4D and S5D for the Marcksl1 KO experiments. These images clearly indicate the same consistent region was sampled for all experimented zebrafish – that is the anterior of the caudal trunk region just behind the yolk extension. Clear indication of this has now been made in the amended manuscript and can be found here:

Page 7, Lines 5 to 7:

“All vessel flow and lumen diameter analysis performed for imaging experiments were taken from the pooled-averages of vessels in the anterior caudal trunk region (just behind the yolk extension; see Fig A – E of S2 Text) for each respective experiment group.”

3. In Table 2, are the listed values specifically tuned to align with the experimental range? If so, it would be useful to know which algorithm was used for this purpose and how unique this particular combination of parameters is.

We thank the reviewer for the insightful concern. No optimization algorithm was employed as this is beyond our modeling expertise. Instead, the pressure modifications were manually intervened based on convergent corrections similar to shooting methods of transforming a boundary value problem to an initial (guess) value problem. This clarification is reflected in the manuscript amendment:

Page 6 Line 25 to Page 7 Line 1:

“With regards to the method of obtaining the pressure settings reported in Table B of S1 Text, the primary guiding principle was first to ensure a mass conservation in the network in that the time-averaged flow rates for both plasma and RBC phases matched between the CA and CV. Next, we incrementally shifted pressure ranges over the initial 5s of flow development to match CFD-predicted peak velocities to peak velocities reported in our experimental reference [17]. We systematically reduced or increased the anterior-posterior trunk pressure inputs to minimize the peak velocity discrepancy between CFD results and our experimental reference to levels below 25% (Table C of S1 Text). For example, if the CFD velocities in the CA were 30% higher than the experiment levels, the anterior to posterior pressure drop in the updated pressure input for the two ends of the CA would be reduced by 30%. Further modification to the pressure inputs involve modulating the pulsatility range by modifying P_diastole and P_range while maintaining the time-averaged P in Eq (1).

We cannot claim that the pressure combinations are fully unique in their representation of flow distribution in the network. The absolute pressure ranges are likely not unique but the differential pressure between one region in the network and another is likely unique to the flow profile given. This is because the interconnectedness of ISVs, DLAV, CA and CV will not permit different permutations of CA and CV anterior to posterior differential pressures to provide the same dorsal to ventral differential pressures in ISVs that result in the present ISV flow profiles. We think the more important concern is whether pooled-average flow data is a representative reference to validate predicted CFD results against. At this point, this is a limitation we acknowledge and we have included this commentary in the amended Discussion section:

Page 18, Lines 3 to 16:

“Another limitation is the use of group-average pooled experiment data for flow validation. As shown in Tables E and F of S1 Text (see also supplementary Fig A – E of S2 Text), each pooled group exhibits variation in the peak velocity levels and vessel diameters (Fig 3A – F, Fig 7B) measured. Admittedly, these biological variations make the utilization of group-averaged flow data difficult to match unique morphometric profiles of each individual network where the discrepancy between the models and experiment group average can be as high as 50% (see Table F of S1 Text). This was most apparent in the ISVs and CVs, where the experimental data saw high variance in flow velocities and RBC flux. With regards to the method of obtaining the pressure settings reported in Table B of S1 Text, a major limitation arose from not employing any optimization algorithm to iteratively correct the pressure input as we do not have this modeling expertise. We acknowledge that our approach may not rigorously demonstrate uniqueness of the pressure field distributions but we should not expect pooled-flow data to uniquely match network geometry specific to a representative zebrafish, especially in vessels that do not display strict stereotypical anatomy such as the CVP and DLAV at 2dpf.”

4. The blood flow in DLAV can be towards the posterior or anterior sections. How is the DLAV pressure boundary condition decided in these faces at the two sides if the zebrafish line used for vessel segmentation only represents the vessel structure?

This is a limitation and uncertainty we have to accept that regions near BCs have some misrepresentation. At this moment we are employing DLAV pressures that are midway between the CA and CV levels. This highlights another issue with using pooled-average flow data sets for BC referencing. In situations like these pertaining to structure-dependent flow direction and magnitude, the optimal approach would be to use flow measurements specific to that particular fish sample for that specific region. We fully acknowledge this limitation and have provided such commentary in the amended discussion:

Page 17 Line 33 to Page 18 Line 2:

“While our study increments modeling methods and the understanding of zebrafish flow physiology and morphogenesis, limitations in scope and accuracy remain. One issue is with the representation of flow physics at the DLAV input faces. As DLAV segments serve to complete the aISV and vISV circulatory loops, blood flow follows local lumen connections that may not follow a strict anterior to posterior directional template [12]. Our model overlooks this robust representation of DLAV flow dynamics, particularly at the 1st and 5th ISV units in the model where DLAV pressures enforced by BCs rather than local network connections dictate the flow direction. While we expect this to incur error in levels of local perfusion to specific DLAV segments, we do not expect this to change the general observations of how flow and WSS trends shift in accordance with the hemorheological and morphological alterations applied to experiments and CFD models in our study.”

Minor comments:

5. Some sections of the manuscript require rephrasing for improved clarity and readability. For instance, the explanation "Flow velocities were set at the two periodic faces of the reservoir domains using the velocity at the shared boundary faces with the main network domain" could be made clearer.

We apologize for the poor phrasing. The statement has been reworded while Figure 1B has been modified to aid the clarification of the point made. The amended statement can be found here:

Page 5, Lines 4 to 6:

“Flow at the two periodic faces of each reservoir domain were set by copying the velocity of the adjacent boundary faces in trunk network domain (Fig. 1B: v_R1_ = v_CA1_, v_R2_ = v_CV1_, v_R3_ = v_CV2_) where pulsatile pressure boundary conditions (BCs) were input (Fig. 1A).”

Furthermore, it might be advantageous to relocate the percentage changes in the comparisons to the supplementary material, enabling a focused presentation of the main outcomes in the body of the paper given the volume of the materials being presented.

We thank the reviewer for the advice. As with our response to reviewer 1 who made a similar request, we have avoided indiscriminate quoting of percentages and statistical data. The remaining quantitative metrics presented in the text are limited to comparisons we wish to highlight. With regards to main outcomes of the paper, we provide a more cohesive summary and interpretation of results in the Discussion section. The discussion section has been entirely re-written to achieve this:

Page 15 Line 3 to Page 19 Line 8

6. On Page 7, Line 18, could the authors elaborate on the term 'lumen-area weighted RBC count'? What is the 'area' referred to in this context?

We apologize for the poor phrasing. We have revised this statement for better clarity and also provided further detail of the calculation in supplementary materials:

Page 8, Lines 7 to 9:

“Here, the relative CA hematocrit is the RBC-count divided by average lumen cross-sectional area (for each embryo) and normalized against the maximum level seen in control morpholino injected embryos (Control MO#3 in Fig Aviii of S2 Text).”

Page 8, Lines 16 to 17:

“see Section A of S2 Text for details of hematocrit and flow rate estimation”

Supplementary S2 Text, Section A

7. In Figure 3, both the control and morpholino groups incorporate 7-9 zebrafish, yet it appears that only four from the control group show a higher relative hematocrit in CA. The coloring used for the two groups is identical and might cause confusion; it would be beneficial to differentiate between the groups visually.

We thank the reviewer for the advice, the data has been visually separated between the two groups using different colors, gray for Gata1 MO and white for Control MO. The reviewer is correct in pointing out that 4 Control MO fish appear to have similar hematocrit levels as the Gata1 MO. We are not particularly concerned by this because as we have already pointed out in the previous critique response, our aim is to obtain a wide hematocrit range to obtain the regression data between hematocrit and flow/diameters.

8. For Figure 3F, can the authors specify the method used to measure the vessel diameter in the model, and the number of aISV and vISV included?

We think this question is partially related to the clarification of which location the diameters were measured from. The locations have now been clarified in supplementary figures S1, S2, S3, S4 and S5. With regards to methods, we follow the approach from our earlier paper, reference [17] in the amended manuscript.

This can be found in Page 11, Lines 3 to 6 of the previous manuscript version

And Page 11, Lines 33 to 36 of the latest manuscript revision, reading:

“RBC-perfused lumen diameters of the trunk vascular network were obtained by performing maximum intensity time-stack projections of the RBC trajectories in accordance with the procedure described in Ye and colleagues [17].”

To summarize, this approach obtains lumen profiles using the time-stacked maximum projection of RBC flow in lumen segments. The diameters are given by the full-width half maximum level of the super-gaussian fit to a radial interrogation of the lumen intensity profile.

The flow rate along the dorsal aorta can vary depending on the distal location as some flow diverts through the ISVs, decreasing towards the posterior tail. Similarly, the size of ISVs is dependent on the location along the tail in a developing embryonic zebrafish. What criteria were used to ensure that the vessels originated from a similar region?

As mentioned in the earlier two clarifications, all vessel regions analyzed were the same anterior caudal region just behind the yolk extension. We are well aware of anterior to posterior weakening of flow levels in the aorta and likewise the ISV diameter increase towards the tail. Bearing these in mind we always imaged the consistent caudal region amongst the samples. Proof of this has been provided by the new network images in supplementary figures S1 to S5.

9. What is the rationale for considering the diameter's dependence on the hematocrit as linear while estimating the diameter change in Model 3?

This is based on the regression obtained from experiment, which was found to have statistically significant slope. We pointed out in both the previous version and revised version of the manuscript that the regression analysis suggests morphological adaptation to the hematocrit reduction. In the revised manuscript we interpreted this as a delay in the ISV size expansion that occurs between 2 to 3 dpf.

Text pertaining to this in the previous manuscript can be found in Page 7, Lines 32 to 35:

“Model 3 was devoid of RBCs in the trunk network with 18% reduced ISV diameters. The ISV diameter reduction in model 3 was performed in accordance with the regression model prediction applied to the experimental data shown in Fig. 3A and 3B.”

Text pertaining to this in the revised manuscript can be found in

Page 8, Lines 22 to 24:

“Model NoRBC2 was devoid of RBCs in the trunk network where ISV diameters were reduced by 18% in comparison to WT and NoRBC1. The ISV diameter reduction in NoRBC2 follows the regression model fit based on the diameter-hematocrit correlation observed in the experiment (Fig. 3A and 3B).”

Page 15, Lines 24 to 28:

“Our own experiments with gata1 MO versus control MO zebrafish indicated a morphological adaptation of reduced ISV lumen diameters in the gata1 MO group at 53 to 57 hpf. Since ISV lumen diameters have been reported to increase in tandem with rises in CA and CV hematocrit from 48 to 72 hpf [17], the ISV diameter reduction in gata1 MO zebrafish in our study indicates a similar delay in the remodeling stage for ISVs in hematocrit-reduced zebrafish networks.”

10. Figure 7.C indicates that the low perfusion group has an RBC flux close to zero. Given that RBC trajectories are employed for vessel diameter estimation, has a sufficient number of ISVs from the zebrafish in the low perfusion group been utilized to measure the diameter presented in section B?

There are still vessels with RBC flow, we can see this in supplementary figures S5 but we agree with the reviewer’s insight. Narrower vessels may still have plasma perfusion but too narrow for RBCs to enter. Bearing that in mind, the average diameter in the low group is likely smaller than reported as a result. It is likely that we do not observe a significant difference in diameters between moderate and low perfusion groups because of the dual effects of the narrower ISVs not being represented in the measurements on top of the total number of sampled ISVs in the low group being too few. This however does not alter our interpretation for the totality of our observations that Marcksl1 KO with strong flow reduction phenotyping in ISVs exhibit overall flow reduction in the CA flow provided to the network.

11. Figure 9 appears to be missing panel F.

We apologize for this typographical error. The legends should read E i) ii) ii) iv) and not Ei) ii) F i) ii). This has been amended in the revised manuscript.

Page 34, Lines 16 to 25

“**Fig 9. Hemodynamic alterations arising from network mispatterning in PlxnD1 model**. (see S11 Movie). **(A)** Spatial distribution map of RBC trajectories/velocities at systolic peak. Dashed lines indicate arterial-venous shunts that bypass DLAV connections in the ISV network. **(B)** The flux bypass in ISV-DLAV network in PlxnD1 versus the ISV-DLAV network in WT. **(C)** Comparison of the time-averaged RBC flow rate in ISVs between WT and PlxnD1 models. **(D)** WSS spatial distribution map in WT (Di) and PlxnD1 (Dii) network models. Red bold lines in Dii indicate low-WSS dead flow regions. **(E)** Comparison of the ventral (i) and dorsal (ii) levels of systolic peak WSS between WT and PlxnD1 aISVs, the ventral (iii) and dorsal (iv) levels of systolic peak WSS between WT and PlxnD1 vISVs. Statistical comparisons in E and F were performed using unpaired two-tailed *t* test. **, *p* < 0.01; ****, *p* < 0.0001.”

12. The authors assert that a key advantage of their model is its ability to represent local topologies. Although 3-D modeling of zebrafish tail structures is not a novel concept, a discussion on the in-plane image resolution used for the reconstruction and the number of in-plane pixels along the diameter would likely be vital and beneficial.

We have already provided commentary on the modeling resolution in the grid-sensitivity section in Materials and Methods section. We would prefer not to have this in the Discussion section as it would break the coherent flow of discussion there. In the Materials and Methods subsection “Grid independence testing”, we have provided an accuracy versus simulation time cost matrix for each resolution employed in both a typical CA/DA sized segment and an ISV sized segment:

Page 23 Line26 to Page 24 Line 4:

“To ascertain the performance matrix of accuracy against computing time in our CFD method, we performed grid resolution independence on two reduced domain sets. The first set was a 40 µm long straight vessel segment that was 8 µm in diameter, which represented an ISV-type vessel test segment (Fig. 11 A and B). Applying a pressure drop of 18 Pa from left to right end, the steady state flow solution was obtained under various grid resolutions: very fine (VF) at ∆t_LBM = 0.125 µs, ∆x = 0.125 µm; fine (F) at ∆t_LBM = 0.25 µs, ∆x = 0.25 µm; medium (M) at ∆t_LBM = 0.5 µs, ∆x = 0.5 µm; coarse (C) at ∆t_LBM = 1 µs, ∆x = 1 µm.”

Page 24, Lines 30 to 41:

“Extrapolating the performance matrix to the zebrafish trunk network domain in the main study (5 times larger than the CA/CV test domain), we determined that a 15-day downtime for a 6 – 8 % error tradeoff in the M grid was most feasible for our zebrafish network analysis. An F level grid on the other hand would require a 230 day computing downtime for each main study case analyzed on a 60-thread parallel computing processor (AMD Ryzen Threadripper 3970X CPU). Further grid coarsening to C level on the other hand incurs heavy accuracy issues and poor qualitative representation of RBC deformation behavior. This was apparent in the ISV test where VF, F and M all showed similar parachute folding profiles of the RBCs, unlike the ruffled folds seen in RBCs in the C grid model (Fig. 11A). In the CA/CV test, the RBCs in C grid model had deformation forces 3 – 10 times larger than M and F grids for the same shearing rates applied, thus indicating poor resolution of membrane deformations between the CGSM and LBM via the IBM (Fig. 11C). Subsequently, we employed M level grids in our main study, where ∆t_LBM = 0.5 µs and ∆x = 0.5 µm.”

We cannot dictate for the audience how many grid points are required for the smallest feature size in a network because it depends on the scientist performing the study as to what the level of acceptable error is for the amount of computing resource afforded and based on the research objective they seek to achieve. Based on our own matrix, we think zebrafish network geometries require at least 0.5 µm grid spacing for the computational model to keep prediction errors below 10% in most segments. However, some narrower regions in DLAV segments can be as small as 1 to 2 µm especially if we are modeling 1 to 2 dpf stage where lumen expansion there is just beginning. For such geometry, local grid refinement to 0.125 to 0.25 µm grid spacing may be necessary. That would complicate LBM because multi-grid formulation requires special treatment of the LBM fluxing parameters at grid interfaces where grid resolution changes.

With regards to our confocal imaging settings, the xy-resolution used is 0.325 µm – this has been included in the Models and Methods section of the revised manuscript.

Page 19, Lines 28 to 31:

“Lumen morphology was visualized by fluorescent dextran (introduced into the blood vessels through microangiography) and imaged using a 40X 1.25NA objective lens on an inverted Olympus IX83/CSU-Wi spinning disc confocal microscope (Yokogawa) with a Zyla 4.2 CMOS camera (Andor). Confocal z-stacks were acquired at 0.29 µm intervals and the xy pixel resolution was 0.325 µm.”

**Have the authors made all data and (if applicable) computational code underlying the findings in their manuscript fully available?**

The [PLOS Data policy](https://journals.plos.org/ploscompbiol/s/materials-and-software-sharing) requires authors to make all data and code underlying the findings described in their manuscript fully available without restriction, with rare exception (please refer to the Data Availability Statement in the manuscript PDF file). The data and code should be provided as part of the manuscript or its supporting information, or deposited to a public repository. For example, in addition to summary statistics, the data points behind means, medians and variance measures should be available. If there are restrictions on publicly sharing data or code —e.g. participant privacy or use of data from a third party—those must be specified.

Reviewer #1: Yes

Reviewer #2: Yes

PLOS authors have the option to publish the peer review history of their article ([what does this mean?](https://journals.plos.org/ploscompbiol/s/editorial-and-peer-review-process#loc-peer-review-history)). If published, this will include your full peer review and any attached files.


If you choose “no”, your identity will remain anonymous but your review may still be made public.

**Do you want your identity to be public for this peer review?** For information about this choice, including consent withdrawal, please see our [Privacy Policy](https://www.plos.org/privacy-policy).

Reviewer #1: No

Reviewer #2: No

Figure Files:

While revising your submission, please upload your figure files to the Preflight Analysis and Conversion Engine (PACE) digital diagnostic tool, [https://pacev2.apexcovantage.com](https://pacev2.apexcovantage.com/). PACE helps ensure that figures meet PLOS requirements. To use PACE, you must first register as a user. Then, login and navigate to the UPLOAD tab, where you will find detailed instructions on how to use the tool. If you encounter any issues or have any questions when using PACE, please email us at [figures@plos.org](mailto:figures@plos.org).

Data Requirements:

Please note that, as a condition of publication, PLOS' data policy requires that you make available all data used to draw the conclusions outlined in your manuscript. Data must be deposited in an appropriate repository, included within the body of the manuscript, or uploaded as supporting information. This includes all numerical values that were used to generate graphs, histograms etc.. For an example in PLOS Biology see here:

<http://www.plosbiology.org/article/info%3Adoi%2F10.1371%2Fjournal.pbio.1001908#s5>.

Reproducibility:

To enhance the reproducibility of your results, we recommend that you deposit your laboratory protocols in [protocols.io](http://protocols.io/), where a protocol can be assigned its own identifier (DOI) such that it can be cited independently in the future. Additionally, PLOS ONE offers an option to publish peer-reviewed clinical study protocols. Read more information on sharing protocols at <https://plos.org/protocols?utm_medium=editorial-email&utm_source=authorletters&utm_campaign=protocols>
